# Colchicine initiated before radiofrequency-induced injury attenuates left atrial inflammation, fibrosis, and susceptibility to atrial fibrillation

**DOI:** 10.1093/europace/euag242

**Published:** 2026-09-11

**Authors:** Abir Attia, Charles-Alexandre LeBlanc, Ewen Le Quilliec, Feng Xiong, Martin G Sirois, Jean-François Tanguay, Jean-Claude Tardif, Martin Aguilar, Roddy Hiram

**Affiliations:** Montreal Heart Institute, Faculty of Medicine, 5000 Belanger Street Montreal, Université de Montreal, Montreal, Canada H1T 1C8; Department of Medicine, Faculty of Medicine, Université de Montreal, Montreal, Canada; Montreal Heart Institute, Faculty of Medicine, 5000 Belanger Street Montreal, Université de Montreal, Montreal, Canada H1T 1C8; Montreal Heart Institute, Faculty of Medicine, 5000 Belanger Street Montreal, Université de Montreal, Montreal, Canada H1T 1C8; Department of Medicine, Faculty of Medicine, 146 rue Léo Saignat, Université de Bordeaux, 33000 Bordeaux, France; Montreal Heart Institute, Faculty of Medicine, 5000 Belanger Street Montreal, Université de Montreal, Montreal, Canada H1T 1C8; Montreal Heart Institute, Faculty of Medicine, 5000 Belanger Street Montreal, Université de Montreal, Montreal, Canada H1T 1C8; Department of Pharmacology and Physiology, Université de Montreal, Montreal, Canada; Montreal Heart Institute, Faculty of Medicine, 5000 Belanger Street Montreal, Université de Montreal, Montreal, Canada H1T 1C8; Department of Medicine, Faculty of Medicine, Université de Montreal, Montreal, Canada; Montreal Heart Institute, Faculty of Medicine, 5000 Belanger Street Montreal, Université de Montreal, Montreal, Canada H1T 1C8; Department of Medicine, Faculty of Medicine, Université de Montreal, Montreal, Canada; Montreal Heart Institute, Faculty of Medicine, 5000 Belanger Street Montreal, Université de Montreal, Montreal, Canada H1T 1C8; Department of Medicine, Faculty of Medicine, Université de Montreal, Montreal, Canada; Montreal Heart Institute, Faculty of Medicine, 5000 Belanger Street Montreal, Université de Montreal, Montreal, Canada H1T 1C8; Department of Medicine, Faculty of Medicine, Université de Montreal, Montreal, Canada

**Keywords:** Catheter ablation, Radiofrequency Cauterization, Atrial fibrillation, Atrial inflammation, Colchicine

## Abstract

**Aims:**

Atrial fibrillation (AF) is the most frequently diagnosed cardiac arrhythmia. Catheter ablation using radiofrequency cauterization (CA-RFC) performed in the left atrium (LA) aims to isolate electrical sources of AF. This intervention is accompanied by frequent AF recurrences affecting 10% of patients at 30 days and 50% at one-year post-procedure. It has been suggested that inflammation could contribute to AF-recurrence post-CA-RFC. Given its anti-inflammatory and cardioprotective properties, colchicine has been proposed as a potential treatment to prevent AF recurrence post-CA-RFC.

**Hypothesis:**

RFC can lead to atrial fibrosis, inflammation, and new-onset AF, which may be mitigated by colchicine.

**Methods and results:**

Male and female Wistar rats (225–275 g) were divided into four groups: Sham or RFC, with or without colchicine (0.25 mg/kg/8 h, intraperitoneally) starting 8 h before surgery until D1 post-procedure. Sham animals underwent surgery without RFC. Electrophysiology and echocardiography assessments were performed on days 1 (D1) and 3 (D3) post-RFC. Colchicine significantly lowered RFC-induced AF vulnerability, improved LA conduction velocity, reduced LA fibrosis, and attenuated LA inflammation post-procedure. Colchicine also prevented RFC-induced CD11c^+^ macrophages while promoting CD206^+^ recruitment in the LA.

**Conclusion:**

Colchicine started before RFC may be an innovative strategy to prevent the development of LA arrhythmogenic substrate post-procedure.

## Graphical Abstract

**Graphical Abstract:**
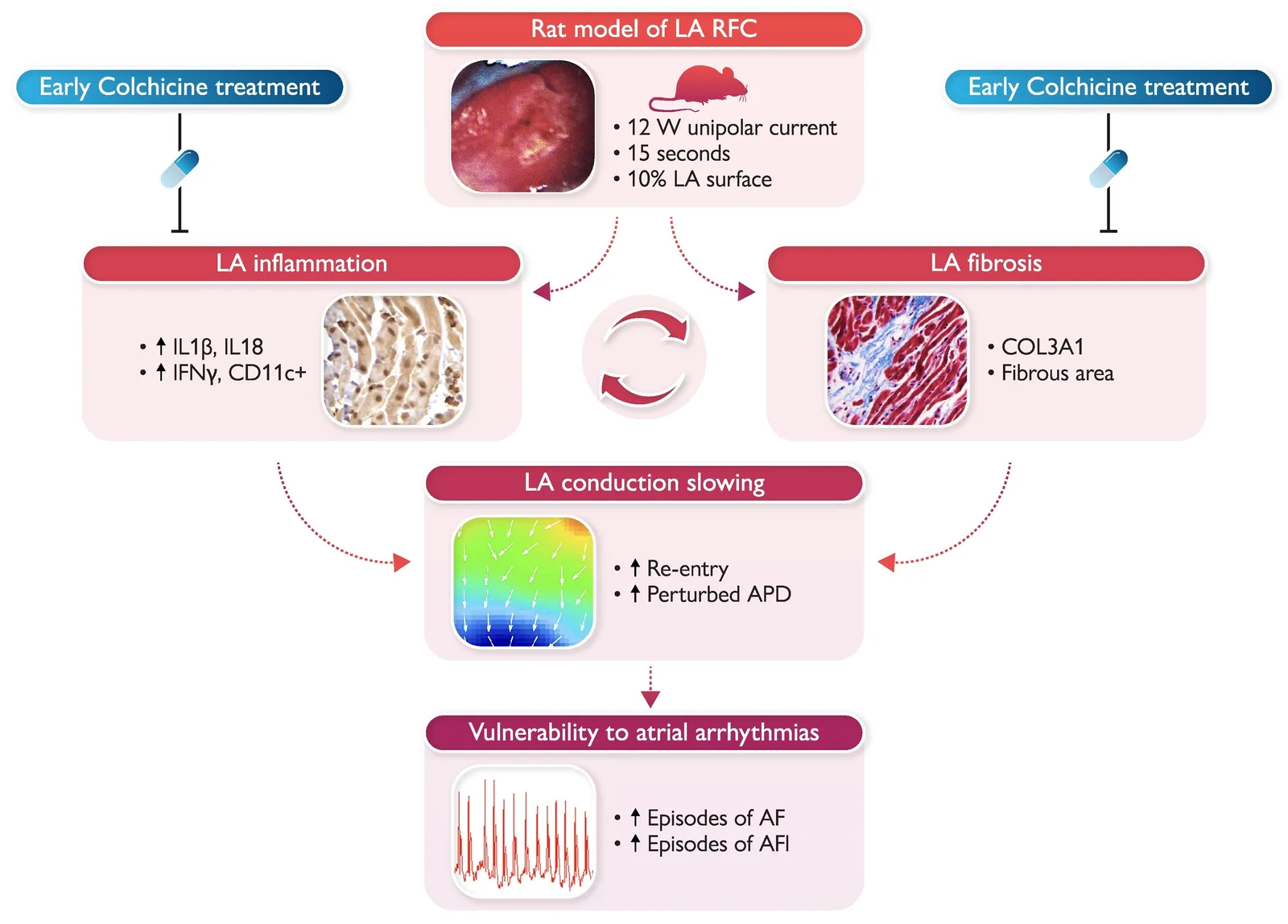
RFC might generate LA inflammation and fibrosis involved in the induction of slowed conduction velocity, abnormal ADP and re-entrant circuits that may be responsible for new-onset AF and AFl post-procedure. Colchicine treatment starting before RFC has the potential to attenuate RFC-induced inflammation and fibrosis, which decreases the vulnerability for cardiac arrhythmias post-RFC. AF, atrial fibrillation; AFl: atrial flutter; APD, action potential duration at 80% of depolarization; COL: collagen; IL, interleukin; IFN*γ*: interferon-gamma; LA, left atrium; RFC: radiofrequency cauterization

What’s new? Collectively, our results support the idea that:

- Inflammation induced by radiofrequency cauterization (RFC) constitutes a major driver of the arrhythmogenic substrate.,
- Early anti-inflammatory intervention—initiated prior to RFC—may be critical to maximizing therapeutic efficacy.
- Prophylactic administration of colchicine may mitigate AF recurrence.
- Colchicine might have a beneficial role as an adjunctive preventive strategy in the peri-ablation period.

## Introduction

Atrial fibrillation (AF) is the most common cardiac arrhythmia, with a global prevalence of above 50 million people.^1,2^ Ageing and comorbidities such as obesity, diabetes, hypertension, and myocardial infarction (MI), are important risk factors for AF.^3,4^ Over the past three decades, basic and clinical studies have substantially advanced our understanding of the pathophysiology of AF, leading to the development and optimization of antiarrhythmic drugs and other therapeutic strategies for rhythm control and patient management.^5–7^

Among the most efficient anti-arrhythmic approaches available, catheter ablation (CA) by radiofrequency cauterization (RFC) is a minimally invasive procedure that uses heat delivered via a catheter to ‘destroy’ electrically archaic areas of the heart tissue causing arrhythmias.^8,9^ CA-RFC generates physical scars acting like a barrier to block faulty electrical signals and restore normal rhythm.^10^ Unfortunately, 10% of patients treated with CA-RFC are affected by AF recurrence 1-month post-procedure, and this incidence can reach 50% at 1 year.^11,12^ Mounting evidence suggests that RFC might cause local inflammation and fibrosis in the atrium which could contribute to AF recurrence during the ‘blanking-period’ post-RFC.^13,14^

Colchicine is an anti-inflammatory medication that has emerged as a potential beneficial strategy to prevent cardiovascular events post-surgery.^15,16^ Colchicine reduces systemic inflammation by lowering pro-inflammatory cytokines like IL-1β.^17,18^ Colchicine acts by binding to tubulin, causing microtubule depolymerization, which disrupts inflammatory cell functions such as intracellular transport, motility, exocytosis, and phagocytosis.^19^ Some but not all of the randomized clinical trials have shown that colchicine reduced early and mid-term AF recurrences compared with placebo in patients undergoing CA-RFC and was associated with improved physical and psychological quality-of-life scores.^20–24^

We hypothesized that LA RFC provokes early-stage inflammation and fibrosis responsible for vulnerability to AF, and that colchicine started before the intervention might attenuate such arrhythmogenic substrate.

The main objectives of this study were to: i) evaluate AF inducibility in a rat model of LA RFC; ii) describe whether colchicine can reduce RFC-induced LA inflammation and fibrosis; and iii) identify whether biological sex-related differences exist in RFC-induced cardiac arrhythmias and in responses to colchicine.

## Methods

### Animal groups

**Figure 1:**
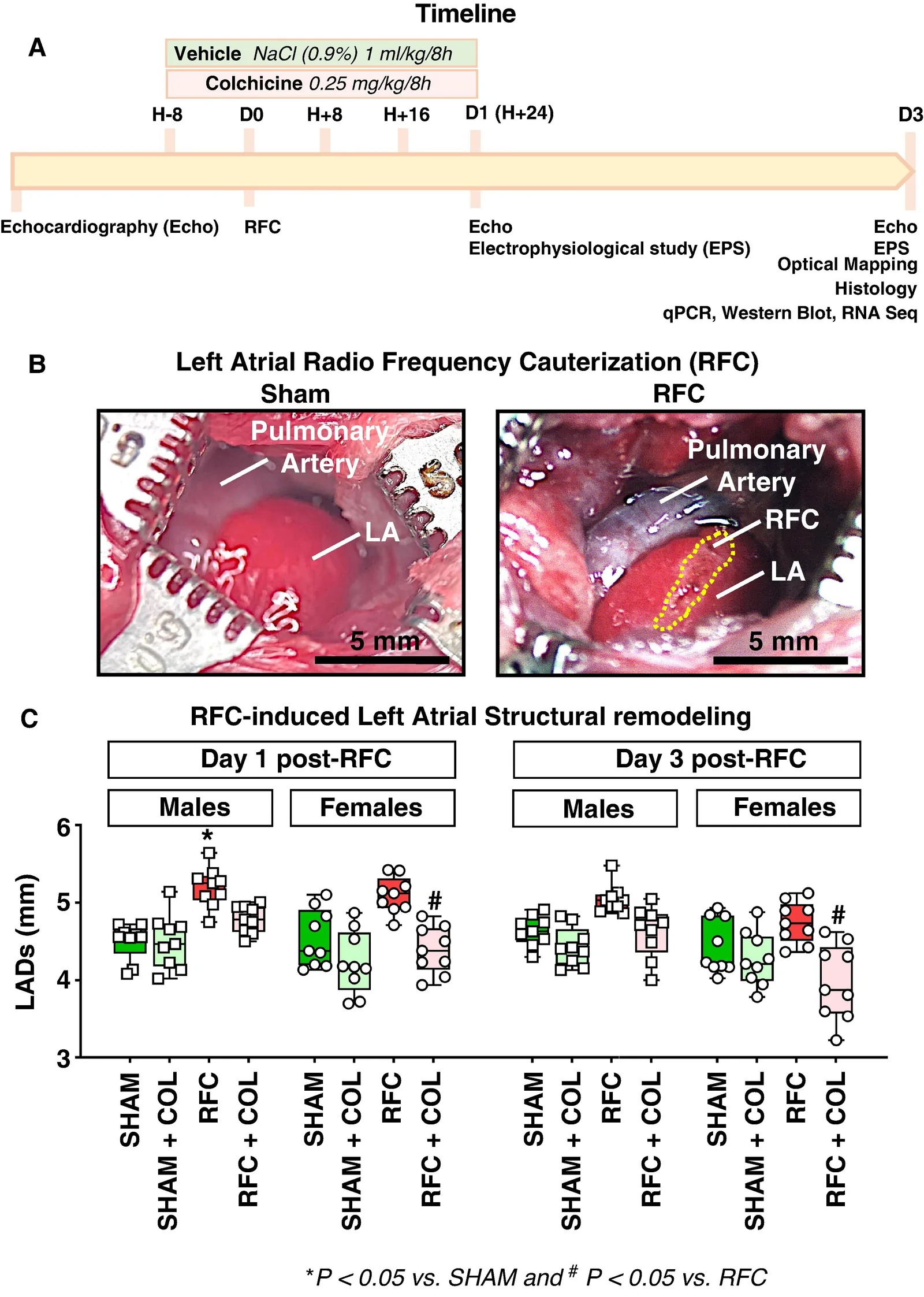
(*A*) Schematic of the chronology of the execution of experiments from beginning of colchicine treatment H-8 before RFC, until euthanasia at D3 post-RFC. (*B*) Representative pictures of open-chest microsurgery performed in sham (left picture) and RFC (right panel) *in vivo*. Scale bars: 5 mm. (*C*) Left atrial dimension at end-systole (LADs) obtained by echocardiography and expressed in mm, in sham, sham + COL, RFC, and RFC-COL rats at D1- and D3 post-RFC. (Statistical analysis: Data were normally distributed as assessed by Shapiro-Wilk test. Two-way ANOVA was performed followed by Tukey correction. Homogeneity of variance was assessed using the Brown–Forsythe test. In cases of heteroscedasticity, Welch’s correction or non-parametric analyses were applied as appropriate to assess significance of differences between the groups Each point results from an individual animal. *n* = 9 rats per group; * *P* < 0.05; *** *P* < 0.01 vs. SHAM and ^###^  *P* < 0.01 vs. RFC).

The study design, protocols, and procedures used in this article were approved by the Animal Ethics Committee of the Montreal Heart Institute, in accordance with the Canadian Council on Animal Care and the NIH Guide for the Care and Use of Laboratory Animals (protocol number: 2023-3230:2022-48-03). Seventy two (72) adult male and female Wistar rats of 8 weeks weighing 225–275 g (Charles River Laboratories, Montreal, Quebec) were equitably randomized by simple-randomization, to 4 groups: sham, sham treated with colchicine (0.25 mg/kg/8h: Sham + COL), RFC, and RFC + COL (9 rats per group per sex category) (*Figure 1A* and Supplementary material online, *Figure S1*).^25^ All animals were healthy, and no exclusion was applied.

### RFC intervention and thoracotomy

At day 0 (D0), all RFC animals were anaesthetized with continued inhalation of 2–3% isoflurane mixed with 2 L/min O_2_. A dose of 0.03 mg/kg buprenorphine was then administrated subcutaneously. Animals were positioned supine on an inclined intubation platform. The tongue was gently extended with a cotton swab and stabilized against the maxilla using a curved spatula. Endotracheal intubation was performed using a 16G angiocatheter mounted on a blunt-stylet introducer, which was advanced into the trachea before removal of the stylet. Correct tube placement was verified by observing normal respiratory movements and condensation on a metal spatula or mirror positioned at the cannula hub. Following successful intubation, animals were transferred to a surgical station in the supine position on a temperature-controlled heating pad and connected to a ventilator (Harvard Apparatus® model 683) to initiate mechanical ventilation at a tidal volume of 2.0–2.5 mL and 70–80 breaths/min. Proper ventilation was further confirmed by the appearance of air bubbles in a water-filled outflow chamber with each respiratory cycle. After stabilization of breathing, the animal was secured in a supine position with limbs carefully immobilized to optimize surgical access while preserving respiratory function. The thorax was shaved, then 70% alcohol and 2% chlorhexidine were used for disinfection.

Under a sterile environment, a 1–2 cm left thoracic incision was performed and the pectoral muscles were bluntly dissected to expose the rib cage. A thoracotomy was performed between the 4th and 5th left intercostal area to allow access to the thoracic cavity, where the thymus and lungs were gently retracted.^26^

The LA was exposed, allowing the performance of the RFC by application of a unipolar radiofrequency current at 12 W for 15 s (s) using a 1.5-mm-diameter radiofrequency catheter connected to a radiofrequency generator (IBI-1500T8, Irvine Biomedical, Irvine, CA, USA). The appearance of a whitish area at the lesion site was observed and considered a visual indicator of RFC formation. The process was repeated point-by-point in order to occupy about 10% of the LA surface (*Figure 1B*). After RFC, the thoracic structures were repositioned, the lungs reinflated, and the rib cage and soft tissues were closed in layers, under local splash-block lidocaine anaesthesia applied with a syringe, prior to final skin closure with a 5-0 Vicryl suture. Sham animals received the same procedure without RFC.

### Colchicine treatment

Starting 8 h before RFC and continued until D1 post-RFC, sham + COL and RFC + COL animals received intraperitoneal (*i.p.*) doses of colchicine (Sigma-Aldrich, Ontario, CA). Rats from the sham and RFC-only groups received an equivalent volume of vehicle (NaCl 0.9%) (*Figure 1A*). Animals were housed at the MHI animal facility during 3 days with *ad libitum* access to water and food. On D1 and D3 post-RFC, echocardiography and transoesophageal electrophysiological studies (EPSs) were performed *in vivo*, under continuous inhalation of 2%-isoflurane mixed with 2 L/min O_2_ for anaesthesia.

On D3, rats were euthanized by exsanguination while under anaesthesia with continued inhalation of anaesthesia (3% isoflurane and 2 L/min O_2_). The freshly excised heart was immediately perfused under the Langendorff system for *in situ* optical mapping. Left atrial (LA) tissues were dissected, snap-frozen, and preserved in liquid nitrogen (N_2_) or formalin-fixed and paraffin-embedded for *in vitro*, histological, and biochemical analyses (see Supplementary material online, *Figure S1*). Each experimental set included an equal number of rats from each group to minimize potential batch effects and confounders. Aware of randomization, Charles-Alexandre LeBlanc performed animal distribution, and all subsequent experiments and analyses were performed by the other students and research personals blinded to experimental groups.

### Echocardiography

Baseline echocardiography was performed on each rat D-1 before RFC, and subsequent echocardiography was performed on each animal at D1 and D3 post-RFC. For each ultrasound session, animals were weighed anaesthetized by inhalation of 2%-isoflurane, 2 L/min O_2_. Acquisition of echocardiograms was performed using a phased array 10S probe (4.5 to 11.5 MHz) in a Vivid 7 Dimension system (GE Healthcare Ultrasound, Horten, Norway) as previously described.^26,27^ To evaluate the left side of the heart, a long-axis parasternal view in 2D M-mode was applied by positioning the ultrasound probe at the level of aortic valve, to acquire the LA dimensions at end-diastole and systole (LADd and LADs) expressed in mm, the LA area (LAAd and LAAs) expressed in mm^2^, and fraction shortening (LA FS) expressed in percent (%).

An apical four-chamber view providing optimal visualization of the LA was acquired. Maximum LA area and length were measured at end-systole, whereas minimum LA area and length were measured at end-diastole. LA maximum (LAVmax) and minimum (LAVmin) volumes were calculated using the modified area–length method implemented in the EchoPAC PC software (GE HealthCare). LA ejection fraction (LAEF) was then calculated as (LAVmax − LAVmin)/LAVmax × 100%. In addition, we also measured left ventricular (LV), right ventricular (RV), and right atrial (RA) parameters by echocardiography, although the RFC did not induce changes among the groups for these chambers. For the LV, the 2D M-mode was used with parasternal short axis view oriented toward papillary muscle, to determine LV dimensions at end-diastole and systole (LVDs and LVDd in mm), LV anterior wall thickness at end-diastole (LVAWd in mm), the LV fractional shortening (FS in %) and the LV ejection fraction (EF in %). Pulsed wave (PW) Doppler was used for *trans* mitral flow in apical four-chamber view. The RA area and dimension were determined using 2D four-chamber view, and the RV function and structure were quantified using parasternal long axis at the level of aortic valve in M-mode and the RV lateral wall using tissue Doppler imaging. Echocardiographic acquisitions were performed by a single experienced technician using a standardized protocol in accordance with a validated standard operating procedure as previously described.^26,27^ To minimize bias, the operator was blinded to the experimental groups during image acquisition. All images were of high quality and suitable for quantitative analysis. Image analyses were conducted independently by a second technician, also blinded to experimental groups. This standardized and blinded workflow minimized intra- and inter-observer variability. Intra- and inter-observer reproducibility was evaluated in a randomly selected subset of echocardiographic examinations. For intra-observer assessment, the same investigator repeated the measurements after an interval of one week, while blinded to the initial results. For inter-observer assessment, a second investigator independently analysed the same examinations while blinded to the measurements obtained by the first investigator. Agreement was evaluated using intraclass correlation coefficients (ICC) with 95% confidence intervals and Bland–Altman analysis. The mean bias and 95% limits of agreement were calculated. Coefficients of variation were additionally reported.

### Transoesophageal stimulation

At D1 and D3 post-RFC, all animals underwent electrophysiological studies (EPS) performed *in vivo* under isoflurane anaesthesia 2%-isoflurane, 2 L/min O_2_ to determine the vulnerability to cardiac arrhythmias with a transoesophageal 4F quadripolar catheter (2-mm interpolar distance; St. Jude Medical #401993). The stimulation threshold during transoesophageal electrophysiological stimulation in rats was determined by progressively increasing the pacing current until consistent atrial capture was achieved. The minimal current voltage capable of inducing reproducible atrial depolarization was defined as the capture threshold.^28^ Atrial stimulation was performed using twelve pacing bursts of 50-Hz during 3-s applied at a voltage equivalent to 4x-threshold current and 2-ms pulse width. Each pacing burst was separated by periods of 2-s rest periods. When pacing burst provoked a sequence dysrhythmia, the next stimulation was not applied to allow the development and spontaneous self-termination of the arrhythmia, allowing the identification of the type and duration of the arrhythmic episode. AF was defined as ultra-rapid irregular atrial beats (>800 bpm). Atrial flutter (AFl) was defined as a regular atrial tachyarrhythmia (600 and 800 bpm). For each inducible animal, AF duration was estimated as the average duration of all AF episodes induced by transoesophageal catheter stimulation. Acquisition and analysis of surface ECG and catheter signals were performed using Iox2 software (version 2.8.0.13, EMKA Technologies, Paris, France).^29^ ECG and EPS analyses were performed by technicians blinded to the experimental groups and treatment allocation, to minimize potential analytical bias.

### Histological analyses

Rats were anaesthetized using 2–3% isoflurane inhalation, 2 L/min O_2_ at D3 post-RFC. LA samples were freshly excised, and following a 24 h fixation step in 10% formalin, RA were dehydrated by immersion in solutions of increasing alcohol concentrations (70%, 95%, and 100%), followed by xylene, embedded in paraffin, cut in 5 μm transversal sections in the mid-segment (mid-depth); and mounted on charged slides. LA samples were then stained with Masson’s trichrome solution to quantify fibrous content at D3 post-RFC. Immunohistochemistry was performed on formalin-fixed LA samples to detect and localize proinflammatory CD11c+ macrophages (CD11c antibody; catalogue number #BS-1014R; ThermoFisher Scientific, CA) and anti-inflammatory CD206+ macrophages (CD206 antibody; catalogue number #ab64693; ABCAM, Waltham, MA). Image Pro Premier 9.3 Software (MediaCybernetics, MD) was used to detect and quantify LA fibrous-tissue content and automated quantification of inflammation-related cells. The radiofrequency lesion was first identified on Masson's trichrome-stained sections by its characteristic dense collagen-rich scar and disruption of the normal myocardial architecture. The lesion region of interest (ROI) was manually delineated by a blinded observer using these histological landmarks with identical criteria applied to all sections, using the Image Pro Premier 9.3 Software (MediaCybernetics, MD). Histological fibrosis quantification was performed on remote atrial regions defined microscopically as tissue located >100 µm from the visible RFC lesion border, whereas the lesion core and adjacent peri-lesional tissue (<100 µm) were excluded to specifically assess distant LA remodelling at D3 post-RFC. Quantifications were performed by an observer blinded to group identity. Fibrous content was expressed as a percent of the analyses LA surface and targeted inflammation-related cells were expressed as the number of cells/mm^2^.^30,31^

### Optical mapping

Rats were anaesthetised using 2–3% isoflurane inhalation, 2 L/min O_2_ at D3 post-RFC. A freshly excised heart was excised and Langendorff-perfused via the aorta with Krebs solution (10 mL/min; 37°C; 95% O_2_; pH 7.5). After a 20 min period of equilibration, blebbistatin (15-µM) was administered and Di-4-ANEPPS (10 µM. 0.1 mL) (Cedarlan, Burlington, Ontario) and was injected into the circulating Krebs solution. Optical signals were acquired using a high-speed CMOS camera system (SciMedia Ltd., Costa Mesa, CA) at a spatial resolution of 256 × 256 pixels and a frame rate of 1000 frames/s, with recording durations of 5 s per trial. Illumination was provided by an LED light source (X-Cite Xylis, Excelitas Technologies, Waltham, MA) set to 15–60% intensity. A S1-S2 protocol was applied to determine the LA effective refractory period (ERP). S1-S2 protocol consisted in a series of seven S1 pacing at basic CL (BCL) of 150 ms followed by an S2-extrastimulus, decrementing by 1 ms from 80 ms until failure to capture. A 25-Hz stimulation was applied directly on the LA chamber to evaluate AF inducibility on isolated hearts *in situ*. LA conduction velocity (CV) and action potential duration to 80% repolarization (APD_80_) were evaluated at BCLs 250, 200, 150, and 100 ms and calculated with a Matlab® algorithm as previously described.^30–34^ LA free-wall CV was quantified using a multi-step optical mapping analysis pipeline. First, background fluorescence was subtracted from each pixel, and the resulting signal was normalized to its peak amplitude. Activation time at each pixel was defined as the time point corresponding to the maximum first derivative during the action potential upstroke (dF/dt_max_). Activation times from all pixels within the selected region of interest were then assembled to generate isochronal LA activation maps for each propagating wavefront. To facilitate comparison across beats, LA activation maps were normalized by subtracting the earliest activation time in each map. For each preparation, five consecutive paced beats were analysed and averaged to obtain a representative activation-time map. Local propagation vectors were then derived from the spatial gradient of the averaged activation-time map, providing both the direction and magnitude of wavefront propagation. Pixelwise CV was calculated from these local LA activation-time gradients and converted to physical units using the spatial calibration of the optical mapping system. The resulting data were displayed as colour-coded LA CV maps with uniformly distributed activation vectors. Mean CV was defined as the average of all valid pixelwise CV values within the analysed region. No separate lesion mask was applied; accordingly, the reported LA CV values reflect conduction within the mapped field as acquired.^30–34^

### Polymerase chain reaction (PCR)

LA tissue samples were freshly isolated and snap-frozen in liquid-N2. RA1 lysis buffer was used for homogenization of tissue. The Nucleospin RNA II® Kit (Macherey Nagel, Düren, Germany) was used for the extraction of RNAs. Nanodrop-2000 (Thermo Fisher Scientific, Watham, MA) was used to assess the concentration of mRNA, and High-Capacity-cDNA Reverse Transcription Kit (Thermo Fisher Scientific, Waltham, MA) helps to retrotranscribe 250 ng of such mRNA samples to produce complementary desoxyribonucleic acids (cDNAs). The obtained cDNAs were adjusted by dilution to achieve a final concentration of 2 ng/µL.^30,31^

Reverse transcriptase-polymerase chain reaction (RT-PCR) was performed using a StepOnePlus Real-Time PCR System (Thermo Fisher). SYBR green primers were used for interleukin 10 (*Il10*), interleukin 18 (*Il18*), interleukin 1 bêta (*Il1b*), epidermal growth factor (*Egf*), and interferon-gamma (*Ifnγ*). SYBR-green primer sequences (forward and reverse) are provided in Supplementary material online, *Table S1*. Gene expression levels were calculated using the 2^−ΔCt^ method, with hypoxanthine–guanine phosphoribosyltransferase 1 (*Hprt1*) used as the reference gene.

### Protein multiplex bead-based discovery assay

LA samples were homogenized in a lysis buffer containing 20 mM Tris-HCl (pH 7.5), 0.5% Tween 20, 150 mM NaCl, and a protease inhibitor cocktail diluted 1:100. After homogenization, the homogenates were centrifuged at low speed (1000–2000 × g) for 10–15 min at 4°C to separate cellular debris. The supernatant was carefully transferred into a clean polypropylene tube, and the protein concentration was then measured using the Bio-Rad Protein Assay Dye Reagent Concentrate (450 mL; Bio-Rad). Total protein concentrations were normalized by adding lysis buffer to samples with higher protein concentrations.

Protein extracts were subsequently analysed to assess the expression levels of cytokines simultaneously using the Rat Cytokine Array/Chemokine Array 27-Plex Discovery Assay kit (Eve Technologies, Calgary, AB, Canada), according to the manufacturer’s instructions.^35^ This assay is based on Luminex®/Multiplex technology and uses a Millipore assay that includes fluorescent beads, each assigned a specific colour code and coated with antibodies targeting 27 distinct cytokines, including IL10, IL18, IL1β, EGF, and IFN*γ*. LA samples were first incubated with the beads, followed by the addition of biotinylated detection antibodies and subsequent incubation with phycoerythrin-conjugated streptavidin. This procedure enables detection of targeted cytokines using the Bio-Rad BioPlex 200 analyzer, which employs a dual-laser system. The first laser identifies specific cytokines through excitation of the coded beads, while the second quantifies the fluorescence intensity emitted by the phycoerythrin-conjugate, which is directly proportional to the amount of captured cytokines. Each assay cycle used a total of 100 µL of sample’s homogenate, performed in quadruplicate to ensure reproducibility, with a maximum coefficient of variation of 4%.^35^

### Statistical analysis

Normality of distribution was assessed using the Shapiro-Wilk test. Non-normally distributed continuous data or data for which normality could not be assessed were tested by Kruskal-Wallis. For categorical outcomes, including AF inducibility, Fisher's exact test was used. When multiple pairwise Fisher's exact tests were required, *P*-values were adjusted using the Holm method to control the family-wise error rate. Comparisons of only 2 groups of data were performed using non-paired Student’s t-tests. Multiple-group continuous outcomes were analysed using two-way analysis of variance (ANOVA) including the factors RFC, colchicine treatment, and their interaction, followed by Tukey-adjusted *post hoc* comparisons when appropriate. Linear mixed-effects models were used to analyse parameters measured repeatedly such as CV and APD_80_ at various BCL during optical mapping, where fixed effects included RFC, colchicine treatment, BCL, and their interaction terms, whereas animal identity was included as a random effect to account for within-subject correlation. When a significant interaction was detected, pairwise comparisons between experimental groups at each BCL were performed using Tukey-adjusted multiple comparisons. In the absence of significant interactions, only significant main effects were interpreted. For factorial analyses, the significance of interaction effects was evaluated before interpretation of main effects. Pairwise *post hoc* comparisons were performed only when supported by a significant interaction or a significant overall group effect.

Homogeneity of variance (homoscedasticity) was evaluated using Brown–Forsythe testing. When unequal variances were detected, Welch-corrected analyses were applied when appropriate to assess significant differences between the groups. Results are presented as Box-and-Whisker plots showing each point (representing individual animal), the mean (cross symbol [+]), the median (horizontal line inside the box), the first quartile (Q1: lower side of the box), the third quartile (Q3: upper side of the box), the minimum value (segment below the box), and the maximum value (segment above the box). Statistical significance was defined as two-tailed *P*-values < 0.05. A sample size of 9 animals per group was estimated to provide approximately 80–90% statistical power to detect large effects and test the study hypothesis.

## Results

### Effects of RFC and colchicine on LA function and structure

Inter- and intra-observer analyses of echocardiographic measurements demonstrated excellent reproducibility. For LADs, the intra-observer ICC was 0.98 (95% CI, 0.95–0.99), with a mean Bland–Altman bias of 0.0016 mm and 95% limits of agreement from −0.183 to 0.180 mm. The inter-observer ICC was 0.98 (95% CI, 0.97–0.99), with a mean bias of 0.0194 mm and 95% limits of agreement from −0.119 to 0.158 mm (see Supplementary material online, *Table S2*).

Compared to baseline echocardiography measurements at D-1 and sham rats at D1 and D3, RFC induced increases of LA systolic dimensions as assessed by LADs (D1: males), LAAs (D1 and D3: males and females), and LAVs (D1 and D3: males) (*Figure 1C* and Supplementary material online, *Figures S2*, *S3*, *S4B* and *S5B*). Colchicine normalized LA systolic dimensions with significance compared to RFC, mainly in females at D1 and D3 (LADs, LAAs, and LAVs) (*Figure 1C* and Supplementary material online, *Figures S2*, *S3*, *S4B* and *S5B*). Please note that we provide LA dimensions at end-diastole to ensure consistency and transparency across animals and groups; however, current echocardiographic standards and recommendations assess LA maximal size at end-systole (as described above). Therefore, the reported diastolic measurements (see Supplementary material online, *Figures S2A*, *S4A* and *S5A*) may underestimate the true LA size and thus may not fully reflect the extent of atrial enlargement. In terms of LA function, we observed that LA EF and LA FS were not significantly affected by RFC (see Supplementary material online, *Figures S5*).

### Effects of colchicine on RFC-related AF inducibility and ECG modifications *in vivo*

**Figure 2:**
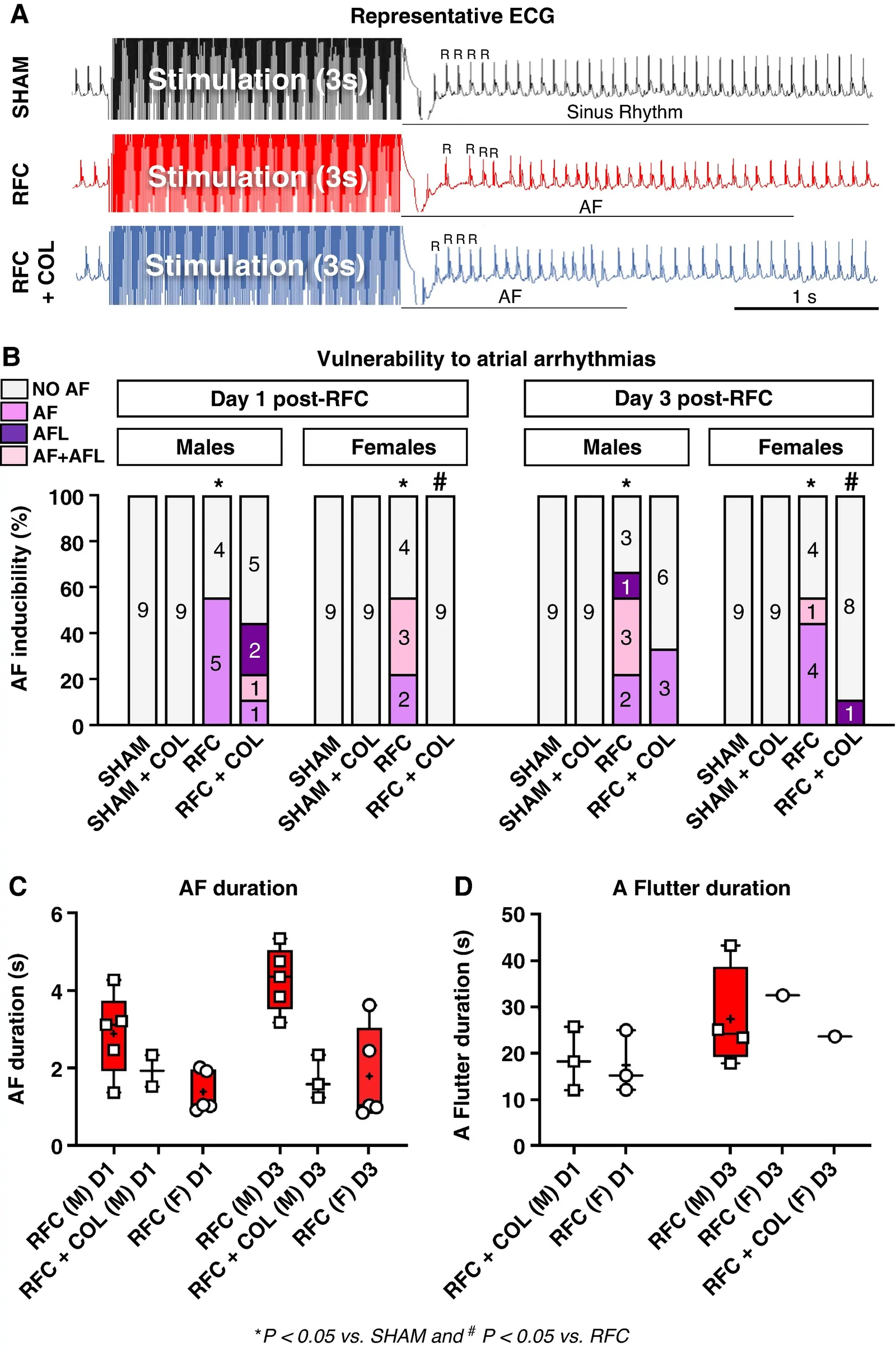
AF vulnerability. (*A*) Representative ECGs showing sinus rhythm, 3 s burst stimulation, and post-induction sinus rhythm or induced atrial fibrillation (AF) episode in sham (dark), RFC (red), or RFC + COL (blue) rats. Scale bar: 1 s. (*B*) Inducibility of AF and AFl during transoesophageal EPS *in vivo*. (*C*) Mean duration of induced AF in inducible RFC-rats. (*D*) Mean duration of induced AFl in inducible RFC-rats. (Statistical analysis: Data were normally distributed as assessed by Shapiro-Wilk test. B: Fisher’s exact test. C and D: Two-way ANOVA was performed followed by Tukey correction. Homogeneity of variance was assessed using the Brown–Forsythe test. In cases of heteroscedasticity, Welch’s correction or non-parametric analyses were applied as appropriate to assess significance of differences between the groups. Only statistically significant pairwise comparisons following significant interaction effects are indicated in the figures. Each point results from an individual animal. *n* = 9 rats per group; * *P* < 0.05 vs. SHAM and ^#^  *P* < 0.05 vs. RFC).

Representative ECGs are shown in *Figure 2A* for Sham, RFC and RFC + COL animals. Male RFC rats at D1 (5/9) and D3 (6/9) were more vulnerable to AF compared to sham animals (0/9) following transoesophageal stimulation *in vivo* (*Figure 2B*). Similarly, female RFC rats at D1 (5/9) and D3 (5/9) were more vulnerable to AF compared to sham animals (0/9) (*Figure 2B*). Colchicine prevented AF vulnerability in females at D1 (0/9) and D3 (0/9) in RFC + COL compared to RFC-only animals, and the benefit was not statistically significant in males (*Figure 2B*). RFC induced AFl in 3/9 females at D1, 1/9 female at D3, and 4/9 males at D3, while 3/9 RFC + COL male rats at D1 and 1/9 RFC + COL female at D3 had AFl compared to no AFl in sham rats (*Figure 2B*). No significant difference was observed among the groups in terms of AF and AFl durations (*Figure* *2C* and *D*). ECG variables, including PR, QRS, QT, and RR intervals, were not statistically significantly different between the experimental groups (see Supplementary material online, *Figure S7*–*S9*). The *P*-wave duration was decreased in male RFC rats at D3, when compared with sham animals, and colchicine treatment prevented this effect in RFC + COL rats (see Supplementary material online, *Figure S7A*). Notably, irrespective of experimental group, females generally exhibited shorter *P*-wave durations and PR intervals than males, consistent with a sex-related difference independent of the experimental intervention.

### Effect of colchicine on RFC-induced left atrial fibrosis

**Figure 3:**
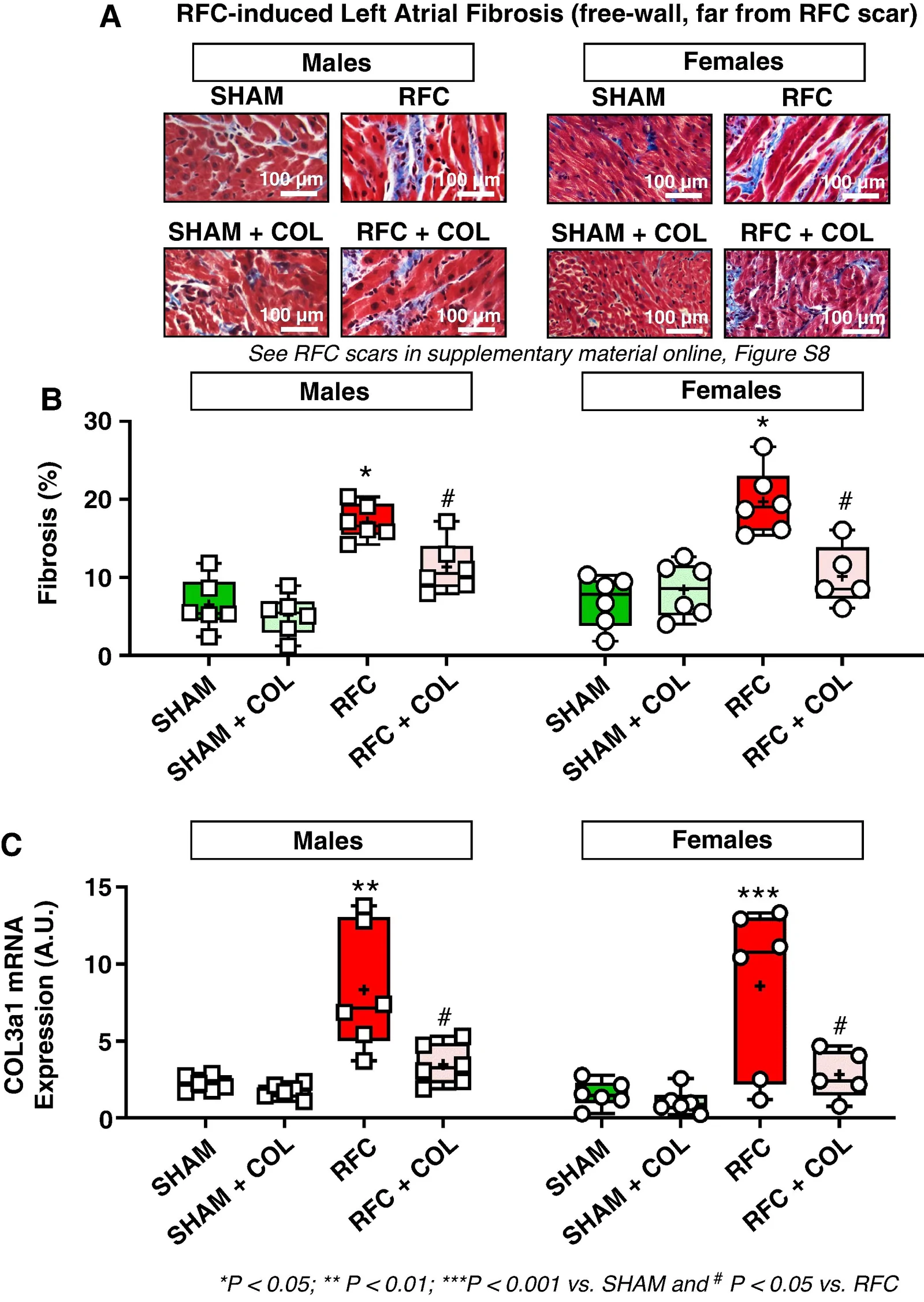
Left atrial fibrosis in remote area, far from RFC scar. (*A*) Representative transverse histological sections of the left atrium (LA) stained with Masson’s Trichrome solution at D3, in male and female sham, sham + COL, RFC, and RFC + COL rats. Scale bars: 100 µm. (*B*) Quantification of the LA fibrous content expressed as a percent of the LA-surface. (*C*) Gene-expression level evaluated by RT-qPCR analysis for *Col3a1* in LA from sham, sham + COL, RFC, and RFC + COL rats. (Statistical analysis: B and C: Data were normally distributed as assessed by Shapiro-Wilk test. Two-way ANOVA was performed followed by Tukey correction. Homogeneity of variance was assessed using the Brown–Forsythe test. In cases of heteroscedasticity, Welch’s correction or non-parametric analyses were applied as appropriate to assess significance of differences between the groups Each point represents results from an individual animal. *n* = 6 rats per group; *P < 0.05; ** *P* < 0.01; ***P < 0.001 vs. SHAM and ^#^  *P* < 0.05 vs. RFC).

RFC increased LA fibrosis in male and female rats compared to sham, as assessed by Masson’s trichrome staining in the area of RFC scar (see Supplementary material online, *Figure S10A* and *B*) and in the remote LA free wall (*Figure* *3A* and *B*). Colchicine reduced fibrosis in the LA free wall (*Figure* *3A* and *B*) but not in the area where RFC was applied (see Supplementary material online, *Figures S10A* and *B*). RFC-induced increase of LA mRNA expression of collagen COL3a1 was prevented by colchicine in male and female RFC + COL rats at D3 (*Figure 3C*)

### Impact of colchicine on RFC-induced LA electrophysiological remodelling *ex vivo*

**Figure 4:**
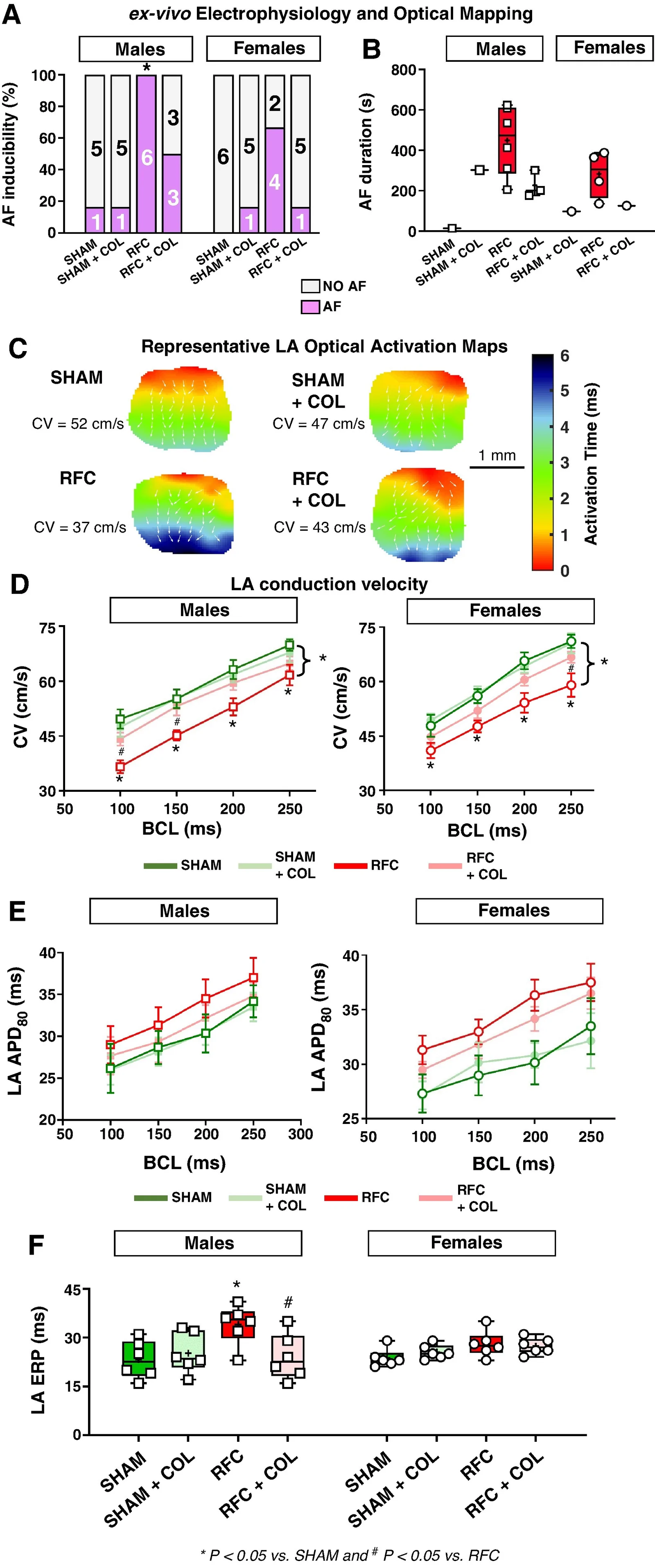
Left atrial optical mapping. (*A*) Inducibility of AF during left atrial (LA) pacing of Langendorff-perfused hearts *in situ*. (*B*) Mean duration of induced AF in male and female inducible sham, sham + COL, RFC, and RFC + COL rats. (*C*) Representative LA activation maps at BCL 100 ms. Scale bar: 1 mm. (*D*) LA conduction velocity at BCL 100, 150, 200, and 250 ms in male (left panel) and female (right panel) rats. (*E*) LA action potential duration at 80% repolarization (APD80) in male (left panel) and female (right panel) rats. (*F*) LA effective refractory period (ERP). (Statistical analysis: Data were normally distributed as assessed by Shapiro-Wilk test. A: Fisher’s exact test. D and E: Linear mixed-effects model including RFC, colchicine treatment, BCL, and their interactions as fixed effects, with animal identity as a random effect, followed by Tukey-adjusted pairwise comparisons where performed; F: two-way ANOVA followed by Tukey correction was applied. Homogeneity of variance was assessed using the Brown–Forsythe test. In cases of heteroscedasticity, Welch’s correction or non-parametric analyses were applied as appropriate to assess significance of differences between the group. Each point results from an individual animal. *n* = 6 rats per group; * *P* < 0.05 vs. SHAM and ^#^  *P* < 0.05 vs. RFC).

In accordance with the electrophysiological data obtained *in vivo*, burst-pacing of the LA performed at D3 *in situ* on freshly isolated and Langendorff-perfused hearts provoked significantly more episodes of AF in male RFC rats (6/6) compared to 1/6 male sham rats (*Figure 4A*). AF episodes occurred in 4/6 female RFC animals which was not significantly more elevated than the 0/6 female sham animals (*Figure 4A*). In addition, 3/6 male and 1/6 female RFC + COL rats were vulnerable for AF, a ratio not significantly different than those observed in sham nor RFC rats (*Figure 4A*). Note that because AF was induced in only one animal in certain groups, the number of inducible rats was insufficient to allow reliable statistical comparison of AF episode duration between groups. Consequently, AF duration analyses were considered descriptive only (*Figure 4B*). No sex-based difference was observed in terms of CV; hence, one representative activation map is provided per group (*Figure 4C*). CV measured at multiple basic cycle lengths (BCL) was analysed separately in males and females using a two-way mixed-effects model fitted by restricted maximum likelihood, with the experimental group as the between-subject factor, BCL as the repeated within-subject factor, and animal identity as the random effect. The model included group, BCL, and group-by-BCL interaction effects. Tukey-adjusted pairwise comparisons were performed between prespecified groups at each BCL. CV in LA was slowed in male and female RFC rats compared to sham animals, and this abnormality tended to be improved by colchicine with significance at BCL 100 and 150 in males and BCL 250 in females (*Figure* *4C* and *D*). Evaluation of group-effect revealed that APD_80_ was globally not significantly modified by RFC compared to sham (*Figure 4E*). The increase in LA ERP induced by RFC in male rats compared to sham was prevented by colchicine in RFC + COL rats (*Figure 4F*).

### Effects of RFC and colchicine on pro- and anti-inflammatory genes and proteins in LA

**Figure 5:**
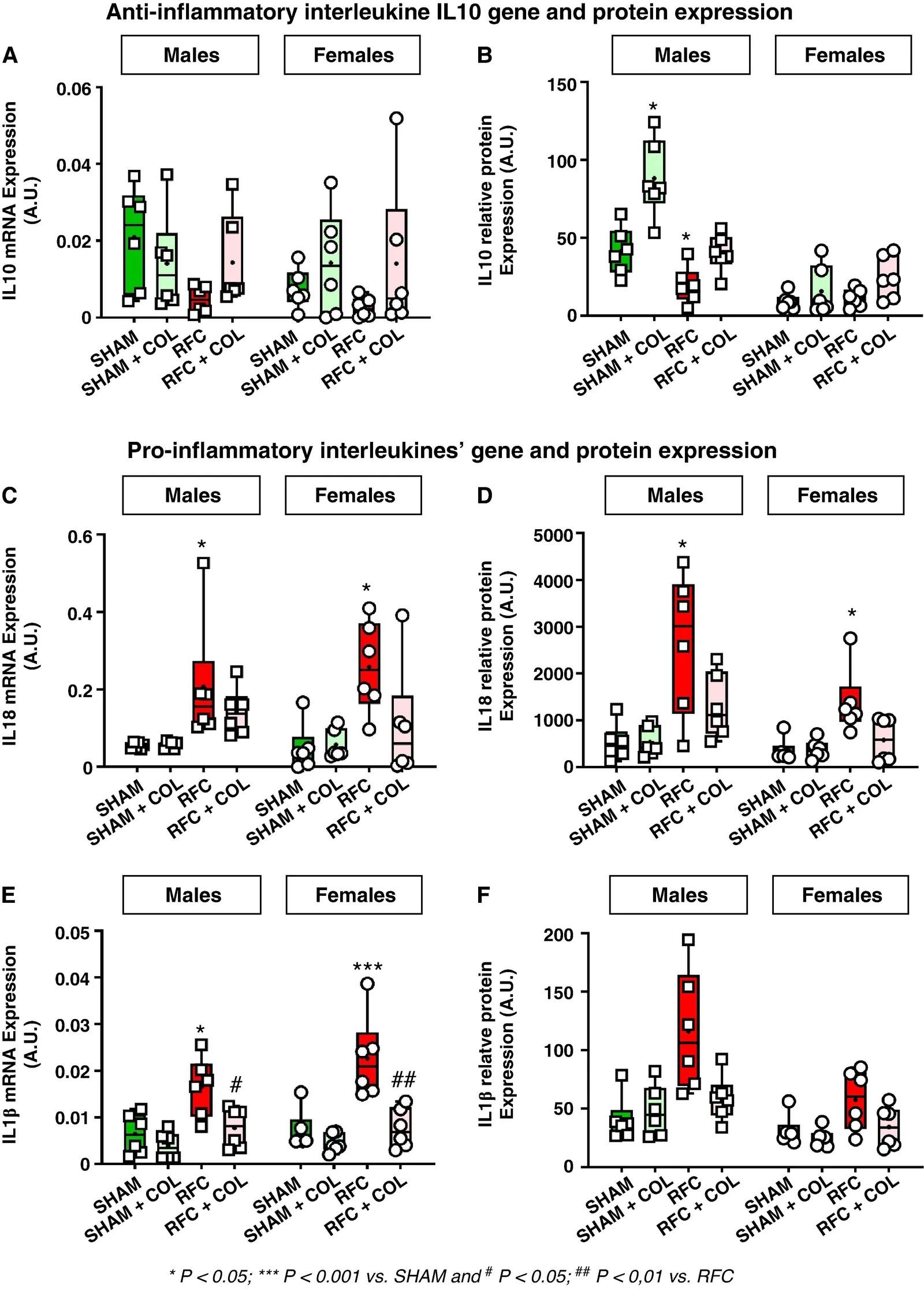
Expression of selected genes and proteins. Gene-expression level (left panels) and protein-expression level (right panels) analysed at D3 post-RFC for anti-inflammatory interleukin (*A* and *B*) IL10, and pro-inflammatory interleukins (*C* and *D*) IL18, and (*E* and *F*) IL1β in LA from male and female sham, sham + COL, RFC, and RFC + COL rats. (Statistical analysis: Normality of distributions was assessed by Shapiro-Wilk test. Normally distributed data (*E*) were analysed by two-way ANOVA followed by Tukey test, and non-normally distributed data (*A–D* and *F*) were analysed by mixed-effect model and Kruskal-Wallis followed by Dunn’s tests. In addition, Brown-Forsythe and Welch ANOVA tests were applied evaluate homo- and heteroscedasticity and assess significance of differences between the groups. Each point represents the level of expression from an individual animal. *n* = 6 rats/group; * *P* < 0.05; *** *P* < 0.001 vs. SHAM and ^#^  *P* < 0.05; ^##^  *P* < 0,01 vs. RFC).

**Figure 6:**
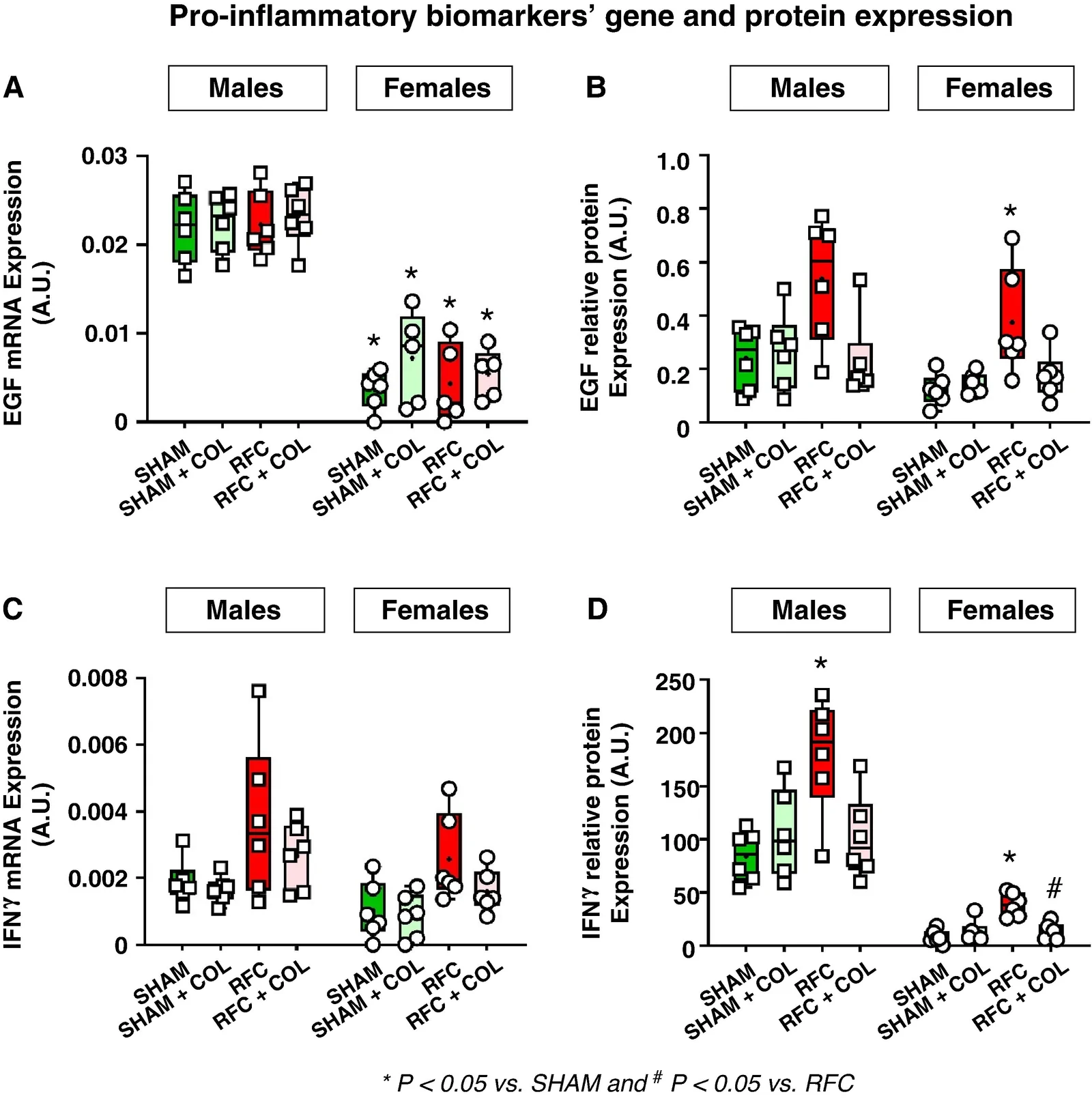
Expression of selected proinflammatory genes and proteins. Gene-expression level (left panels) and protein-expression level (right panels) analysed at D3 post-RFC for (*A* and *B*) EGF and (*C* and *D*) IFN*γ* in LA from male and female sham, sham + COL, RFC, and RFC + COL rats. (Statistical analysis: Normality of distributions was assessed by Shapiro-Wilk test. Normally distributed data (*A*) were analysed by two-way ANOVA followed by Tukey test, and non-normally distributed data (*B–D*) were analysed by mixed-effect model and Kruskal-Wallis followed by Dunn’s tests. In addition, Brown-Forsythe and Welch ANOVA tests were applied to evaluate homo- and heteroscedasticity and assess significance of differences between the groups. Each point represents the level of expression from an individual animal. *n* = 6 rats/group; * *P* < 0.05 vs. SHAM and ^#^  *P* < 0.05 vs. RFC).

The whole-LA mRNA and protein expressions of selected interleukins and biomarkers involved in anti-inflammatory (IL10) and pro-inflammatory (IL18, IL1β, EGF, IFN*γ*) responses were evaluated at D3 by qPCR and protein multiplex discovery-assay respectively (*Figures 5* and *6*). Compared to sham animals, IL10 protein was reduced in male RFC rats and increased in sham + COL rats (*Figure 5B*). IL18 mRNA and protein expressions were increased in male and female RFC rats compared with sham and were not significantly affected in RFC + COL rats (*Figure* *5C* and *D*). IL1b mRNA was higher in male and female RFC rats compared to sham, and this increase was prevented by colchicine in RFC + COL rats (*Figure* *5E* and *F*). LA mRNA levels of *Egf* were lower in females compared to males (*Figure 6A*), and RFC significantly increased EGF protein levels in females; however, this effect was abolished by colchicine treatment, which maintained EGF expression at sham levels (*Figure 6B*). RFC significantly increased IFNy protein expression in both sexes, while colchicine prevented this effect and preserved IFNy mRNA and protein levels at sham values (*Figure* *6C* and *D*).

### Effects of RFC and colchicine on LA recruitment of CD11c+ and CD206+ positive macrophages

**Figure 7:**
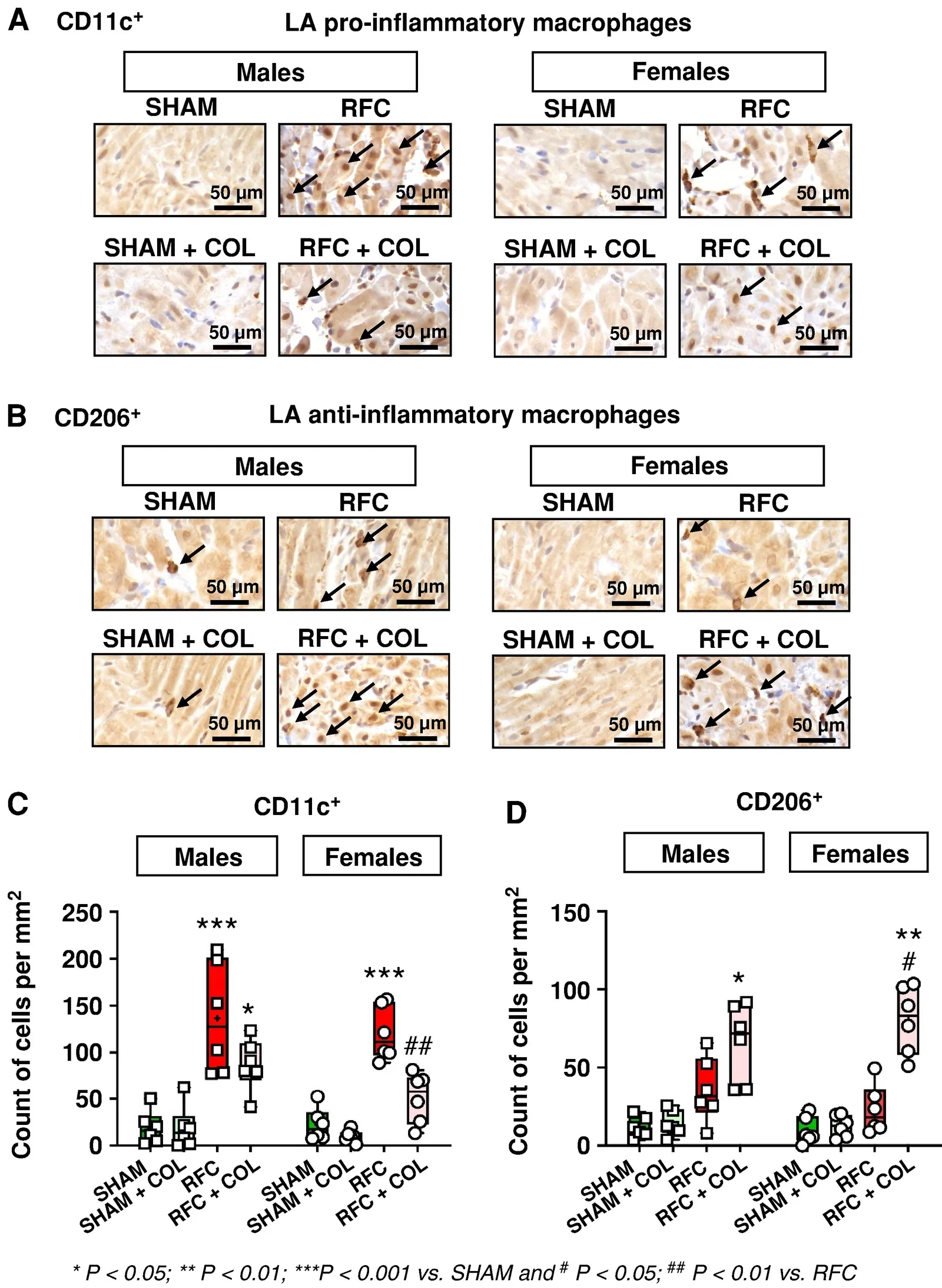
Left atrial expression of CD11c and CD206 positive inflammatory macrophages. Immunohistochemical staining (brown) of (*A* and *C*) proinflammatory macrophage marker CD11c, and (*B* and *D*) anti-inflammatory macrophage marker CD206 in LA at D3 post-RFC. Arrows indicate representative macrophages. Scale bars: 50 µm. Graphs showing the quantification of (*C*) atrial CD11c positive cells and (*D*) CD206-positive cells per mm^2^ in LA. (Statistical analysis: Data were normally distributed as assessed by Shapiro-Wilk test. Comparisons were performed using two-way ANOVA followed by Tukey correction. In addition, Brown-Forsythe and Welch ANOVA tests were applied to evaluate homogeneity of variances and to assess significance of differences between the groups. Each point represents the level of expression from an individual animal. *n* = 6 rats/group; * *P* < 0.05; ** *P* < 0.01; ***P < 0.001 vs. SHAM and ^#^  *P* < 0.05; ^##^  *P* < 0.01 vs. RFC).

Immunohistochemistry revealed that the recruitment of proinflammatory CD11c^+^ macrophages was significantly increased in the LA from male and female RFC rats compared to sham animals (*Figure* *7A* and *C*).

Colchicine reduced CD11c^+^ macrophages in the LA in female RFC + COL rats compared to RFC only animals (*Figure* *7A* and *C*). CD206^+^ anti-inflammatory macrophages in the LA were significantly increased in female RFC + COL rats compared to RFC only animals (*Figure* *7B* and *D*).

## Discussion

### Main findings

In this study, we found that LA RFC can generate LA remodelling including atrial fibrosis and inflammation, slowed conduction velocity, and increased AF vulnerability. Colchicine treatment starting before the procedure might contribute to prevent AF inducibility by attenuating inflammation by reducing the expression of LA pro-inflammatory biomarkers such as IL1β and IFNγ (*Graphical Abstract*).

### RFC-induced arrhythmogenic structural remodelling and biological sex-based differences

In this study, we employed a model of RFC applied on the LA in rats to replicate structural changes observed in humans following CA-RFC.^36^ While CA-RFC can effectively isolate pre-existing electrical arrhythmogenic AF substrates, the cauterization injury itself may also promote local inflammation and fibrotic remodelling, potentially creating a new pro-arrhythmogenic substrate that could contribute to later AF recurrence.^37^ In this context, our experimental model was intentionally designed to apply focal RFC injury to an otherwise healthy LA in order to specifically characterize RFC-induced atrial remodelling and its association with AF susceptibility, independently of pre-existing atrial disease. Notably, longitudinal studies in humans have reported reductions in LA volume of 15–20% one year after successful CA-RFC, with smaller LA volumes associated with higher AF recurrence, particularly beyond 12 months.^38,39^ In contrast, during the 4 weeks post-ablation, it has been described that the procedure could provoke atrial swelling.^40^

Interestingly, in our model, following RFC, structural and molecular remodelling was also observed in atrial regions remote from the primary RFC lesion, suggesting that RFC-induced injury may trigger diffuse atrial remodelling beyond the direct site of thermal damage. This phenomenon may involve propagation of inflammatory signalling, oxidative stress, fibroblast activation, autonomic imbalance, secondary extracellular matrix remodelling, and development of fibrosis throughout the atrial tissue.^41^ The partial attenuation of these changes by colchicine in our study supports the contribution of inflammation-mediated mechanisms in the development of remote atrial remodelling following RFC.

Sex-related differences in atrial remodelling remain underexplored. Clinical data suggest that women exhibit more pronounced structural remodelling, higher AF recurrence rates post-CA-RFC, and slower CV than men, independent of atrial dilation.^42^

Studies have reported that RFC triggered a robust inflammatory response, activating pro-fibrotic pathways, promoting fibroblast-to-myofibroblast differentiation, and increasing collagen deposition.^43^ This aligns with meta-analytic evidence linking LA fibrosis to post-CA-RFC AF recurrence.^44^ Our data partially support these observations. Results trended to highlighting augmentations in LA dilation (LADs, LAAs, LAVs) post-RFC. Colchicine seemed to prevent RFC-induced LA dimension changes with statistically significant efficacy in females (*Figure 1C* and Supplementary material online, *Figures S2*–*S5*). In contrast, our histological analysis confirmed significant increase in LA fibrosis and augmentation of biomarkers, including COL3A1, following RFC in both sexes (*Figure 3*). Interestingly, in our study, although colchicine reduced remote fibrosis in both sexes, a reduction in AF inducibility was observed predominantly in females, suggesting that structural remodelling alone may not fully explain the anti-arrhythmic effects of colchicine.

These findings raise the possibility that sex-dependent electrophysiological, hormonal, or autonomic mechanisms may modulate the functional response to colchicine. In particular, differences in atrial refractoriness, conduction properties, inflammatory signalling, and fibroblast activity may contribute to the distinct susceptibility to AF observed between males and females. Further mechanistic studies are warranted to clarify the pathways underlying these sex-specific responses.

### RFC-associated AF vulnerability

Our findings demonstrate that RFC itself can provoke AF vulnerability in a preclinical rat model, even in the absence of prior arrhythmic predisposition. Although clinical AF occurring after cavotricuspid isthmus (CTI) ablation or other atrial ablation procedures is likely influenced by underlying atrial disease,^45^ our findings suggest that radiofrequency injury itself may also contribute to AF susceptibility through inflammation- and fibrosis-associated atrial remodelling. By applying focal RFC to otherwise healthy rat atria, our model was specifically designed to isolate and characterize the direct arrhythmogenic consequences of RFC-induced tissue injury independently of pre-existing atrial pathology.

Mechanistically, AF recurrence is often linked to pulmonary vein reconnections, post-ablation re-entrant circuits, and arrhythmogenic remodelling characterized by extensive atrial fibrosis.^46^ In our model, *in vivo* electrophysiological assessments revealed that 55.56% of male and female rats were susceptible to post-RFC arrhythmias at D1 post-procedure, rising to 66.67% in males and 55.56% in females by D3 (*Figure 2*). Although the precise mechanisms underlying the observed ECG changes need to be further investigated, the normalization of these parameters following colchicine treatment suggests that RFC-induced inflammatory remodelling and associated autonomic or electrophysiological alterations may contribute to these transient conduction and repolarization changes.

Collectively, our data confirm that LA RFC can directly trigger AF, providing an experimental platform for investigating preventive interventions and sex-specific responses in post-ablation arrhythmias.

### RFC-induced left atrial inflammation

In our study, RFC increased gene expression of pro-inflammatory markers IL1β, IL18, and IFNγ, while modulating IL10 (protein) and EGF (gene) levels in a sex-specific manner. RFC also promoted LA recruitment of proinflammatory macrophages CD11c^+^. This result aligns with clinical data implicating elevated IL1β, IL18, and IFNγ in postoperative AF.^47,48^

Histologically, we observed significant LA fibrosis, corroborated by molecular markers such as COL3A1, reinforcing the link between inflammation, fibrotic remodelling, and susceptibility to AF (*Figures 2–7*). Collectively, these findings suggest that post-RFC inflammation is a critical driver of arrhythmogenic remodelling, influencing AF recurrence, and highlighting the importance of targeting inflammatory pathways in therapeutic strategies. Moreover, the potential sex-specific differences in inflammatory (IL10 protein expression and *Egf* gene expression) responses underscore the need for tailored approaches in managing post-CA-RFC AF.

### Impact of colchicine in RFC-induced arrhythmogenic substrate

Inflammation emerges as a central driver of the structural and electrophysiological changes related to cardiac arrhythmias.^49^ Recently, it has been shown that colchicine exerts anti-arhythmic, anti-remodelling effects and infibitory impact on inflammasome activity in a rat model of reduced ejection fraction and myocardial infarction.^50^ In our study, prophylactic colchicine administration prior to RFC significantly reduced AF inducibility. This effect was accompanied by a reduction in COL3A1 expression, attenuated interstitial fibrosis, improved conduction velocity and significantly reducing pro-inflammatory CD11c^+^ macrophages, gene *Il1b* and protein IFNγ, suggesting a dual anti-inflammatory and anti-fibrotic mechanism (*Figures 3–7*).

Accumulating clinical evidence supports the concept that targeting inflammation represents an effective strategy to limit postoperative and post-ablation AF. In the context of cardiac surgery, meta-analyses of randomized controlled trials encompassing 912 patients demonstrated that perioperative colchicine administration reduced the incidence of postoperative AF by 35% over follow-up periods ranging from one to six months.^21,51,52^ Beyond arrhythmia prevention, the COLCOT trial (COLchicine Cardiovascular Outcomes Trial) further established the cardiovascular benefits of inflammation suppression, showing that low-dose colchicine significantly reduced ischaemic cardiovascular events (5.5%) compared to placebo (7.1%) over a median follow-up of 22.6 months.^23^

In terms of CA-RFC, Deftereos *et al.* reported that colchicine administered at a dose of 0.5 mg twice daily significantly reduced AF recurrence at both 3 months (16% vs. 33.5% in controls) and 12 months (31.1% vs. 49.5%), an effect that was paralleled by a reduction in inflammatory biomarkers implicated in AF pathophysiology.^20,52,53^ These findings have been consolidated by subsequent meta-analyses, which consistently confirmed the dual anti-inflammatory and antiarrhythmic effects of colchicine following AF ablation or/and pulmonary vein isolation (PVI) procedures.^53–55^

### Controversial reports on colchicine benefits in the management of post-operative AF

Although the potential benefits of colchicine following AF ablation are suggested,^56^ these conclusions are partly driven by observational and heterogeneous studies. Importantly, randomized clinical trials have yielded conflicting results, with some reporting reduced AF recurrence whereas others failed to demonstrate significant clinical benefit.^24^ Therefore, the potential role of colchicine in the post-RFC ablation setting remains incompletely established and may depend on multiple factors beyond treatment initiation timing alone, including patient substrate characteristics, inflammatory burden, ablation strategy, and treatment duration.

Some studies have reported no significant reduction in AF recurrence with postoperative colchicine following catheter ablation.^24^ These discordant findings likely stem from important methodological differences, particularly regarding treatment timing. In some trials, colchicine was initiated after the ablation procedure rather than prophylactically as performed in our model, thereby limiting its ability to blunt the early inflammatory response that contributes to arrhythmogenic substrate formation. In addition, inconsistent exclusion or adjustment for concomitant antiarrhythmic therapies across colchicine and placebo groups may have confounded outcome interpretation in some trials. Colchicine administration is also frequently associated with gastrointestinal adverse effects, including vomiting, diarrhoea, and dehydration, which can precipitate electrolyte disturbances and increase susceptibility to cardiac arrhythmias, including AF.^57–59^ Consequently, failure to exclude colchicine-intolerant patients may bias efficacy analyses. Notably, in studies reporting colchicine inefficacy, although adverse events were acknowledged—and might themselves trigger AF—methodological adjustments were not systematically implemented to consider these effects. Moreover, these investigations did not distinguish whether newly observed AF episodes in the colchicine group were attributable to arrhythmogenic side effects or represented genuine post–ablation recurrence.

Importantly, no colchicine-related adverse effects were observed in our experimental model, contrasting with human studies where gastrointestinal symptoms and dehydration can precipitate arrhythmias and complicate interpretation.^57^ These data reinforce the importance of early, pre-procedural administration of colchicine to maximize anti-inflammatory benefits and mitigate arrhythmogenic risk.

### Clinical implications

Our findings may have implications for the understanding of arrhythmias, including AF, occurring during the post-ablation ‘blanking period’.^60^ Recent clinical discussions and expert consensus statements suggest that early atrial arrhythmias following AF ablation during the 90 days post-ablation may not necessarily reflect procedural failure but rather transient inflammatory, autonomic, and tissue-healing processes associated with lesion maturation following RFC injury.^45,60^ In this context, the present study supports the concept that RFC itself can transiently create a pro-inflammatory and pro-arrhythmogenic atrial substrate, even in previously healthy tissue. These observations may also be relevant when considering emerging non-thermal ablation technologies, which may induce different degrees of tissue inflammation and remodelling compared with conventional radiofrequency energy.^61–63^

The atrial structural and electrophysiological alterations observed following RFC may also be interpreted within the contemporary framework of atrial cardiomyopathy.^64^ Recent consensus statements define atrial cardiomyopathy as a complex substrate involving structural, architectural, contractile, and electrophysiological remodelling processes that promote atrial dysfunction and arrhythmogenesis.^65^ In our study, RFC-induced inflammation, fibrosis, LA enlargement, and increased AF susceptibility collectively support the concept that focal RFC injury may transiently induce an acquired atrial cardiomyopathy-like phenotype, even in previously healthy atrial tissue (*Figures 1–7*). The partial attenuation of these changes by colchicine further suggests that inflammatory mechanisms contribute importantly to the development and propagation of post-RFC atrial remodelling.

### Limitations and perspectives

#### Duration of study

In this study, we focused on D3 post-procedure, a time point identified in the literature as representing a peak of inflammation in the rat model, thereby justifying the study duration.^66^ To further elucidate the long-term effects of colchicine treatment, future investigations could consider additional time points, such as D7, D14, and D21 post RFC. In addition, the selection of D3 was intended to mimic a clinical scenario in which colchicine is administered for one week following CA-RFC, with subsequent electrophysiological assessment at D90 post-ablation.^52^ This approach considers the 90-day blanking period post-RFC.^14,41,60,61^ However, to comply with the guidelines of the MHI’s institutional animal ethics committee, it was mandatory to adjust the experimental timescale according to prior data indicating that one human year corresponds to approximately 13.8 days in rats.^67^ Consequently, the 90-day clinical study was translated into a 3-day protocol in the rat model.^52^ The experimental 3-day window in rats is an early post-procedural phase relative to the 90-day clinical follow-up period. However, we acknowledge that such temporal extrapolation may not uniformly reflect the kinetics of inflammation, fibrosis, and electrophysiological remodelling across species.^68^ Accordingly, the present study should be regarded as a proof-of-concept, laying the groundwork for future investigations with extended study durations (e.g. D7, D14, D21).

#### Our model of RFC in rat partially mimics the *trans*-septal CA-RFC performed in human

A further limitation of the study is the inability to perform endocardial atrial ablation due to the small size of the rats’ heart, as it is feasible in humans. Therefore, the rat model presented in this paper only partially replicates the clinical procedure, because the CA-RFC was applied to the surface of the LA via open-chest surgery. Technically, the experimental model used epicardial focal LA RFC application rather than clinically relevant endocardial pulmonary vein isolation and therefore only partially reproduces the anatomical and procedural characteristics of human AF ablation. The model was primarily designed to investigate the direct biological consequences of RFC-induced atrial injury in a controlled setting rather than to replicate therapeutic clinical ablation strategies. In addition, residual post-ablation oedema may have contributed to the early inflammatory response observed after RFC; however, oedema was not specifically quantified in the present study, but the occurrence of such condition in response to RFC nourishes the idea that the ablation *per se* might be responsible for local atrial inflammation. Overall, this methodological difference, compared to the procedure in humans, warrants caution in the interpretation of our results.

In the present study, the remote atrial fibrosis was quantified in regions located above 100 µm from the visible radiofrequency lesion border. Although a distance of 100 µm is readily distinguishable under microscopic examination and allowed consistent and reproducible definition of the remote region across all specimens, subtle peri-lesional biological effects extending beyond this boundary could not be completely excluded. Therefore, a limited contribution of local radiofrequency-induced remodelling to the quantified remote fibrosis cannot be completely ruled out.

#### Atrial cell-specific analysis of the inflammatory response to LA RFC

The use of whole LA homogenates provided an integrated assessment of global RFC-induced inflammatory and fibrotic remodelling but did not allow identification of the specific cellular populations contributing to these molecular changes. Although this approach was selected to maximize tissue utilization (rats’ LA is very small and analysable cells after enzymatic isolation is challenging) and adhere to the 3Rs principles by limiting animal use, future studies using cell-specific or spatially resolved analyses will provide additional mechanistic insight into the cellular drivers of RFC-induced atrial remodelling.

#### Colchicine did not provoke human-like adverse effects in the rat model

Colchicine-related adverse effects, such as diarrhoea, vomiting, or abdominal pain,^67,69^ were not observed in our rat model, in contrast to clinical reports in humans. This finding suggests enhanced drug bioavailability in rats, which may have contributed to the observed efficacy in our experimental conditions.

#### Biological sex-based differences in RFC-induced AF and colchicine response

Although incorporating male and female rats is a strength in this study, few clinical trials have rigorously addressed and reported sex-related differences in response to colchicine for AF recurrence post-RFC. Therefore, the observed differences between males and females in our model should be interpreted as hypothesis-generating. Although fibrosis reduction was observed in both sexes, additional mechanisms beyond structural remodelling may contribute to AF susceptibility and could be characterized in future studies. Further investigations specifically designed to evaluate sex-dependent electrophysiological and molecular responses to colchicine in patients are needed. Accordingly, our findings regarding potential sexual dimorphism in this model require further validation in human studies.

## Conclusions

Our study reveals that RFC might provoke slight structural changes accompanied with an inflammatory and fibrous status affecting the LA which generate an arrhythmogenic substrate associated with increased vulnerability for AF and AFl. Colchicine treatment starting before the intervention contributed to prevent AF and AFl inducibility, mainly in female rats, probably by the attenuation of RFC-induced LA inflammation via reduction of tissular expression of biomarkers such as IL1β and IFNγ.

## Supplementary Material

euag242_Supplementary_Data

## Data Availability

All raw data supporting the findings from this study are available from the corresponding authors upon reasonable request.
