# Supplementary material for: Colchicine initiated before radiofrequency-induced injury attenuates left atrial inflammation, fibrosis, and susceptibility to atrial fibrillation
Source: Europace. 2026 Sep 11;28(9):euag242. doi: 10.1093/europace/euag242 (PMC13585461; doi:10.1093/europace/euag242)
Supplement: Supplementary file 1 [file euag242_supplementary_data.pdf]

SUPPLEMENTARY MATERIAL ONLINE

Colchicine initiated before radiofrequency-induced injury attenuates left atrial inflammation, fibrosis, and susceptibility for atrial fibrillation

Abir Attia[1,4], Charles-Alexandre LeBlanc[1,4], Ewen Le Quilliec[1,2], Feng Xiong[1], Martin G. Sirois[1,3], Jean-François Tanguay[1,4], Jean-Claude Tardif[1,4,*], Martin Aguilar[1,4,*], and Roddy Hiram[1,4,*]

[1]Montreal Heart Institute, Faculty of Medicine, Université de Montreal, Montreal, Canada

[2]Université de Bordeaux, Bordeaux, France

[3]Department of Pharmacology and Physiology, Université de Montreal, Montreal, Canada

[4]Department of Medicine, Faculty of Medicine, Université de Montreal, Montreal, Canada

Short Title: Colchicine reduces AF vulnerability post LA radiofrequency cauterization

Category: Research Article

***Corresponding authors**

Address for Correspondence: Dr. Roddy Hiram, Montreal Heart Institute Research Center, 5000 Belanger Street Montreal, Quebec. Canada H1T 1C8

Tel: 514-376-3330 ext.: 5015

email: roddy.hiram@icm-mhi.org

Table of Contents

Table S1 SYBR green primers______page 03

Table S2 Inter- and intra-observer agreement LADs of echocardiography measurement______page 04

Figure S1 Distribution of animals per experiments______page 05

Figure S2 Baseline echocardiography of the Left Atrium______page 07

Figure S3 Representative echocardiography at D1 and D3 post-RFC______page 09

Figure S4 LA dimension______page 11

Figure S5 LA volume ______page 13

Figure S6 LA ejection fraction and fraction shortening ______page 15

Figure S7 Electrocardiogram analyses (P and PR) ______page 17

Figure S8 Electrocardiogram analyses (QRS and QT)______page 19

Figure S9 Electrocardiogram analyses (R-R) and Threshold______page 21

Figure S10 Left Atrial Fibrosis in RFC scar area ______page 23

Online Table S1. SYBR green primers

Gene	Forward primer	Reverse primer
**Anti-inflammatory interleukin gene**		
Il10	GGTGACAATAACTGCACCCAC	TATGTTGTCCAGCTGGTCCT
**Pro-inflammatory interleukins genes**		
Il18	GTGTGCCAGGACACAACAAA	AGCATCATCTTCCTTTTGGCA
Il1b	TAGCAGCTTTCGACAGTGAGG	CCACAGCCACAATGAGTGAC
**Pro-inflammatory biomarkers**		
Egf	GTCCCGTTGAAGACGATCAA	CAGGGTAGAAGACACAGCTTAC
Ifnγ	GTGAACAACCCACAGATCCA	GAATCAGCACCGACTCCTTT

Online Table S2. Inter- and intra-observer agreement of LADs echocardiography measurement

Animal ID	Observer 1, first analysis (in mm)	Observer 1, second analysis (in mm)	Observer 2 Analysis (in mm)
AA04M	4.259	4.234	4.181
AA07M	4.135	4.091	4.192
AA09M	4.082	4.113	4.001
BB03M	4.467	4.495	4.203
BB05M	4.020	4.102	3.907
BB08M	4.133	4.198	4.096
CC02M	5.382	5.404	5.367
CC04M	5.278	5.295	5.255
CC08M	5.311	5.296	5.303
DD02M	4.783	4.861	4.813
DD04M	4.502	4.392	4.498
DD06M	5.006	5.018	4.970
AA04F	4.354	4.513	4.375
AA07F	4.184	4.097	4.113
AA08F	4.203	4.311	4.27
BB01F	4.171	4.208	4.189
BB05F	4.023	4.113	4.075
BB09F	4.178	4.137	4.201
CC01F	5.414	5.219	5.394
CC04F	5.211	5.139	5.178
CC08F	4.942	4.780	4.892
DD02F	4.040	3.910	4.062
DD07F	4.241	4.301	4.298
DD09F	4.380	4.511	4.401
**Intra-observer agreement:** Observer 1, first versus second analysis ICC (95% CI)		**0.98 (95% CI, 0.95 - 0.99)**	
Blant-Alman intra-observer results		Mean bias: **0.0016 mm**<br>Lower 95% agreement limit: **-0.183 mm**<br>Upper 95% agreement limit: **0.180 mm**	
Inter-observer agreement: Observer 1, first analysis versus Observer 2 analysis ICC (95% CI)			**0.98 (95% CI, 0.97 – 0.99)**
Blant-Alman inter-observer results			Mean bias: **0.0194 mm**<br>Lower 95% agreement limit: **-0.119 mm**<br>Upper 95% agreement limit: **0.158 mm**

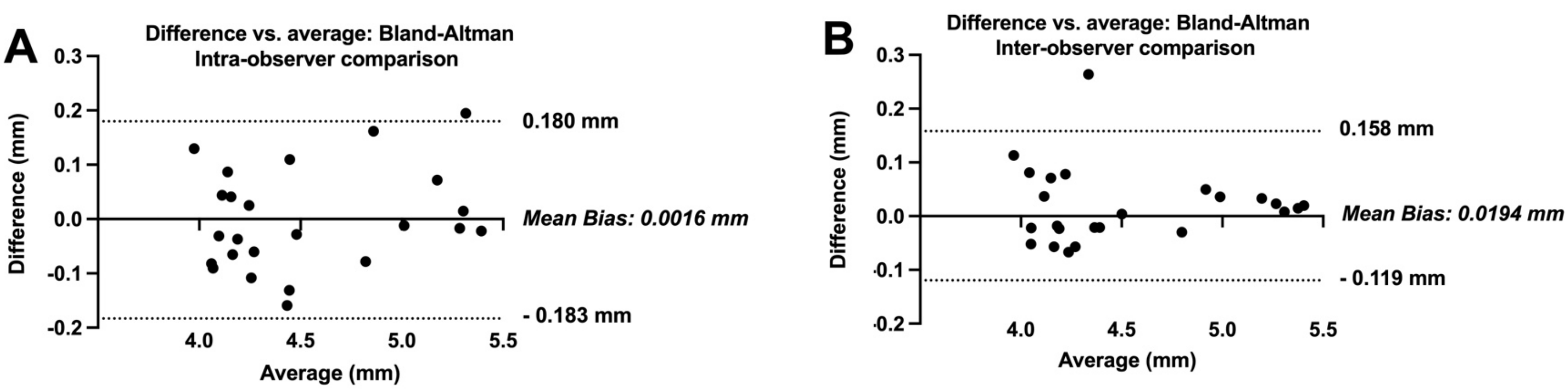

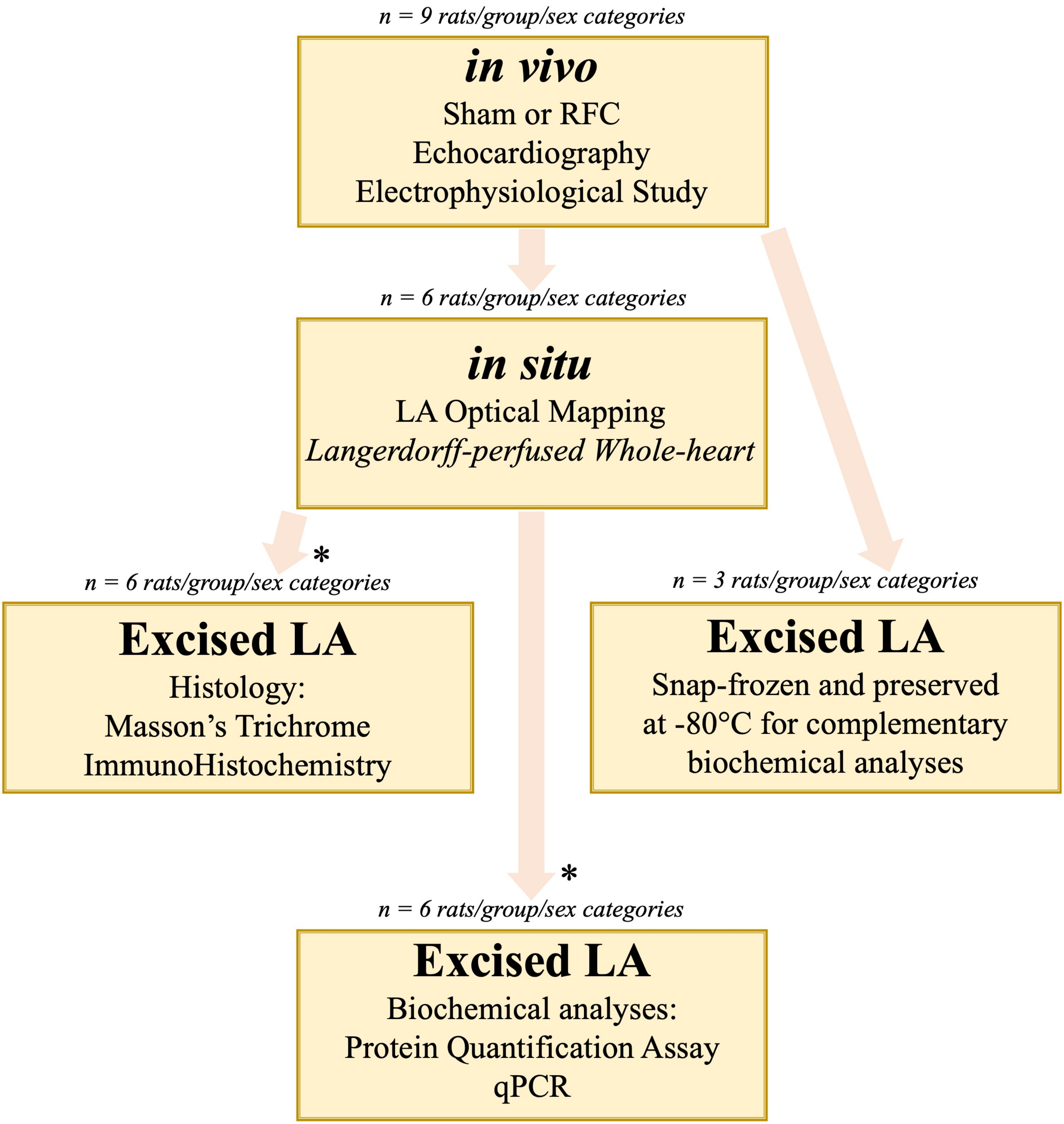

* *LA were excised and split for histological studies (2/3 of the LA surface containing the RFC scar and the remote area) and biochemical analyses (1/3 of the LA remote area)*

Online Figure S1. Distribution of animals per experiments. Precise distribution of animals for each specific experiment. After *in vivo* experiments at D-1 before RFC and at D1 and D3 post-RFC (echocardiography and transesophageal electrophysiological study), hearts were excised at D3 post-RFC for *in situ* optical mapping and dissected for histology, qPCR, and protein quantification assay analyses.

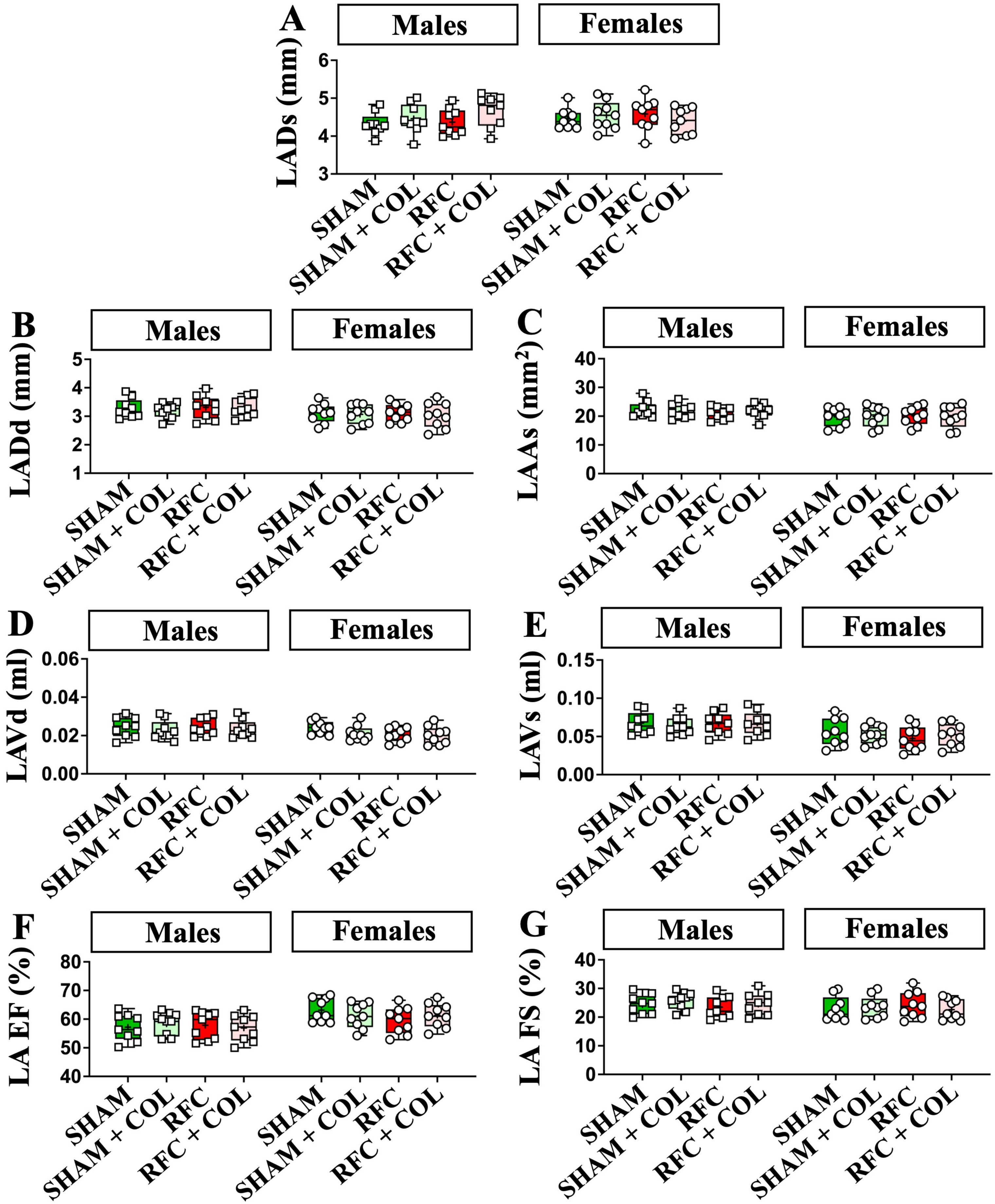

A
Males
Females
LADs (mm)
SHAM
SHAM + COL
RFC
RFC + COL
B
LADd (mm)
C
LAAs (mm²)
D
LAVd (ml)
E
LAVs (ml)
F
LA EF (%)
G
LA FS (%)

Online Figure S2. Baseline echocardiography of the left atrium (LA). Left atrial dimension at end-systole (LADs) (**A**) and end-diastole (LADd) (**B**) expressed in mm. Left atrial area at end-systole (**C**) expressed in mm^2. Left atrial volume at end-diastole (LAVs) (**D**) and end-systole (**E**) expressed in ml. Left atrial ejection fraction (LA EF) (**F**) and fraction shortening (LA FS) (**G**) expressed in percent. Data were obtained by echocardiography in male and female sham, sham + COL, RFC, and RFC-COL rats 1 day before RFC. (*Statistical analysis: Data were normally distributed as assessed by Shapiro-Wilk test. Two-way ANOVA followed by Tukey correction. Homogeneity of variance was assessed using the Brown–Forsythe, and Welch test was applied to confirm no significant differences between the groups Each point results from an individual animal was used to compare the groups. n = 9 rats per group*)

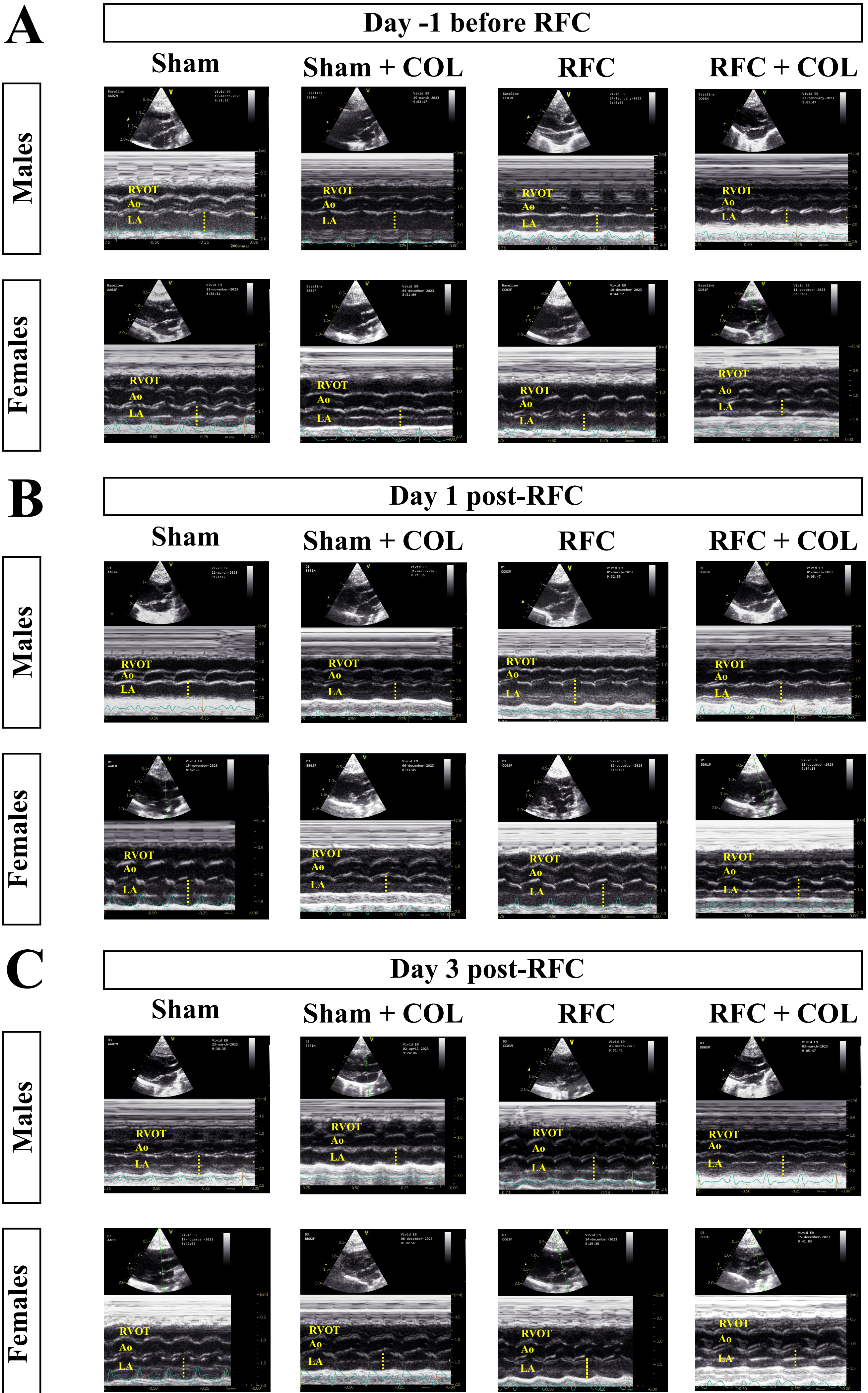
A
Day -1 before RFC
Sham
Sham + COL
RFC
RFC + COL
Males
Females
RVOT
Ao
LA
B
Day 1 post-RFC
Sham
Sham + COL
RFC
RFC + COL
Males
Females
RVOT
Ao
LA
C
Day 3 post-RFC
Sham
Sham + COL
RFC
RFC + COL
Males
Females
RVOT
Ao
LA

Online Figure S3. Representative echocardiography at baseline (D-1), and post-RFC D1 and D3. Pictures obtained by long-axis parasternal view in 2D M-mode at the level of aortic valve, to evaluate the LA dimensions in sham, sham + COL, RFC, and RFC-COL rats at D-1, D1, and D3. Ao: Aorta; LA: left atrium; RVOT: right vernicle outflow tract.

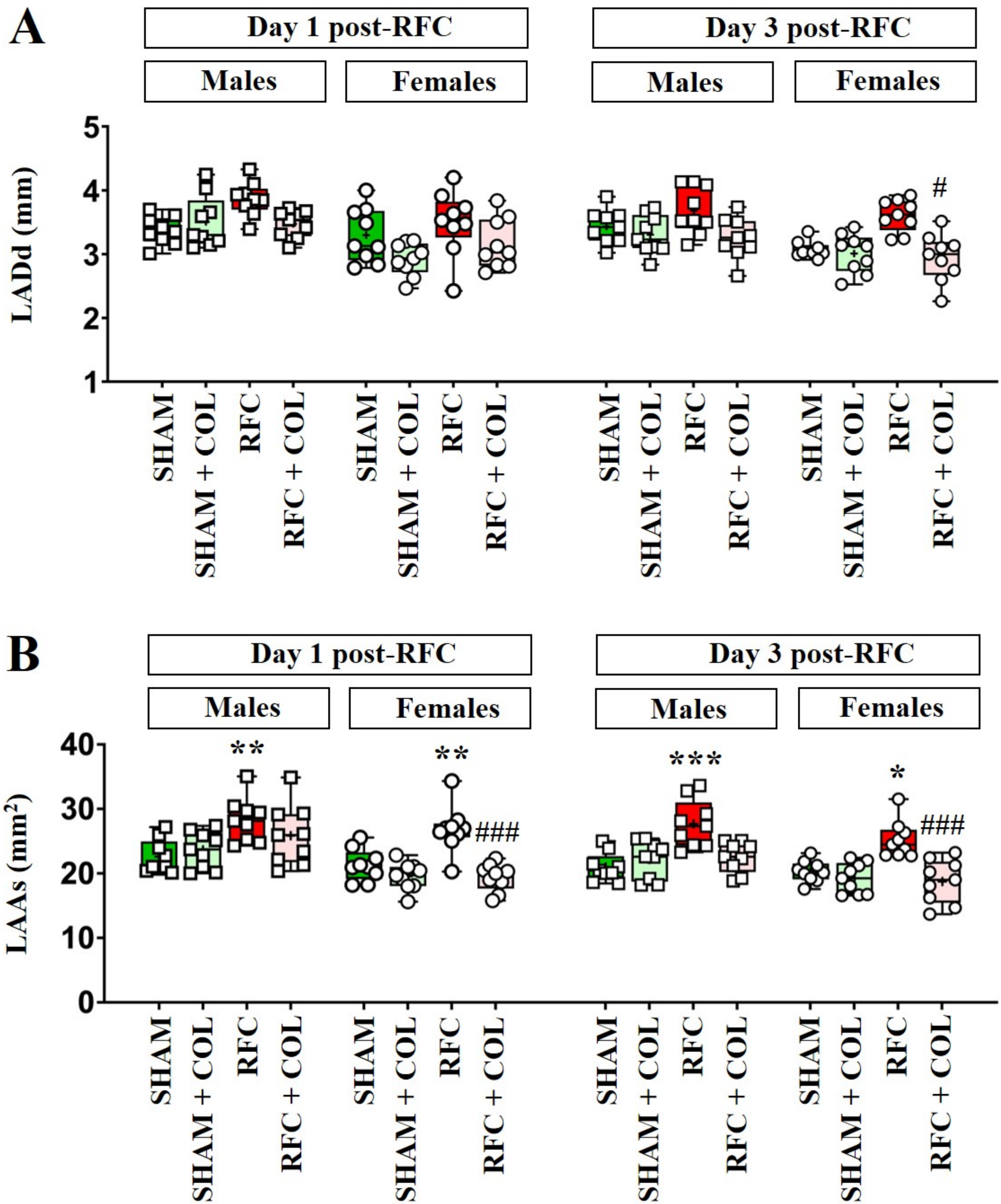

** p<0.05; ** p<0.01; *** p<0.001 vs SHAM and # p<0.05; ### p<0.001 vs RFC*

Online Figure S4. LA dimension. (**A**) Left atrial dimension at end-diastole (LADd) expressed in mm, and (**B**) left atrial area at end-systole (LAAs) in mm^2, obtained by echocardiography, in male and female sham, sham + COL, RFC, and RFC-COL rats at D1- and D3 post-RFC. *(Statistical analysis: Data were normally distributed as assessed by Shapiro-Wilk test. Two-way ANOVA was performed followed by Tukey correction. Brown–Forsythe and Welch tests were used to assess homogeneity of variance and significance of differences between the groups Each point results from an individual animal. n = 9 rats per group; * $p<0.05$; ** $p<0.01$; *** $p<0.001$ vs SHAM and $^{\#}$ $p<0.05$; $^{\#\#\#}$ $p<0.001$ vs RFC).*

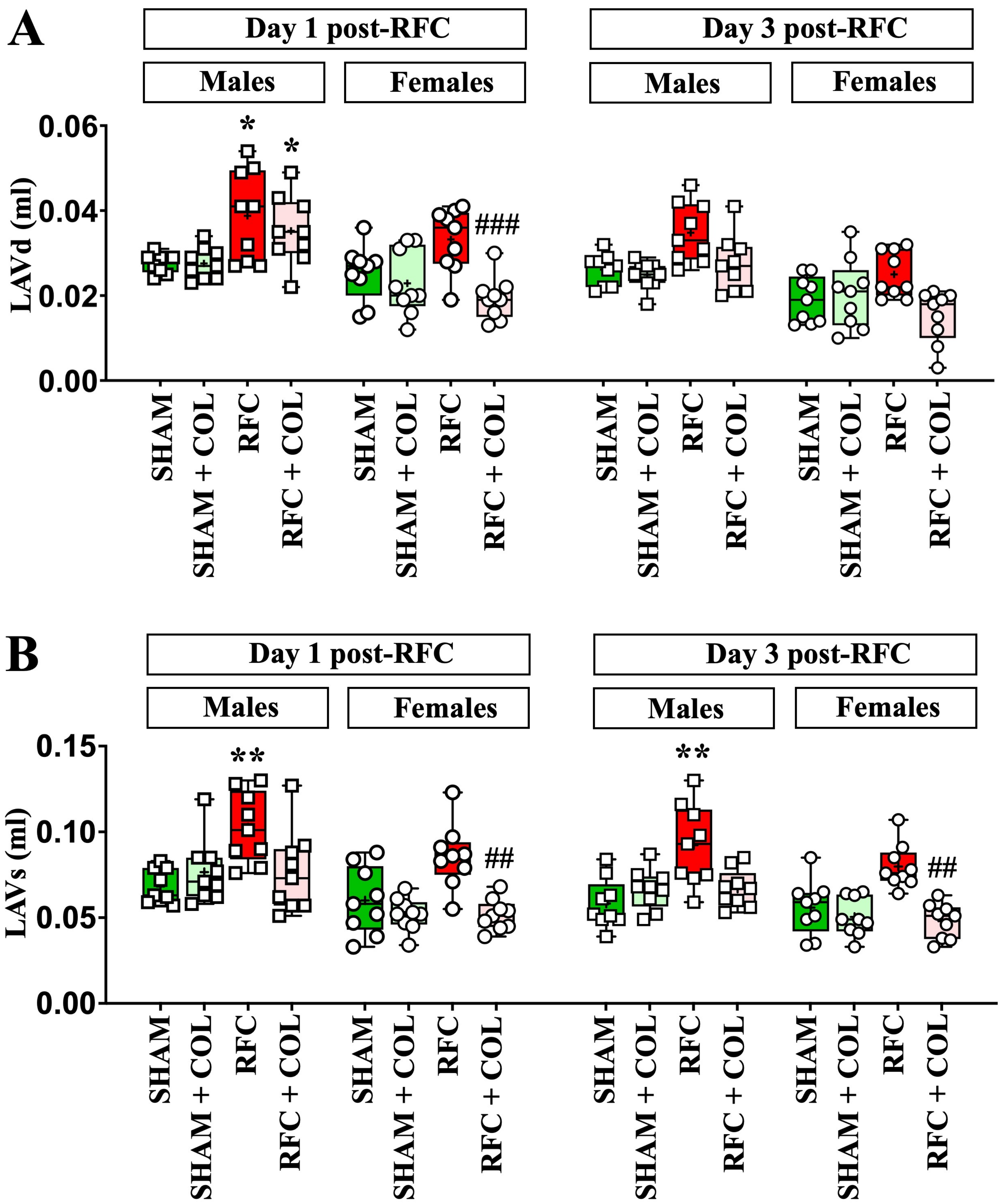

** p<0.05; ** p<0.01 vs SHAM and ## p<0.01; ### p<0.001 vs RFC*

Online Figure S5. LA volume. (**A**) Left atrial volume at end-diastole (LAVd) and (**B**) left atrial volume at end-systole (LAVs) obtained by echocardiography and expressed in ml, in male and female sham, sham + COL, RFC, and RFC-COL rats at D1- and D3 post-RFC. *(Statistical analysis: Data were normally distributed as assessed by Shapiro-Wilk test. Two-way ANOVA was performed followed by Tukey correction. Homogeneity of variance was assessed using the Brown–Forsythe, and Welch test was used to assess significance of differences between the groups Each point results from an individual animal. n = 9 rats per group; * p<0.05; ** p<0.01 vs SHAM and ## p<0.01; ### p<0.001 vs RFC).*

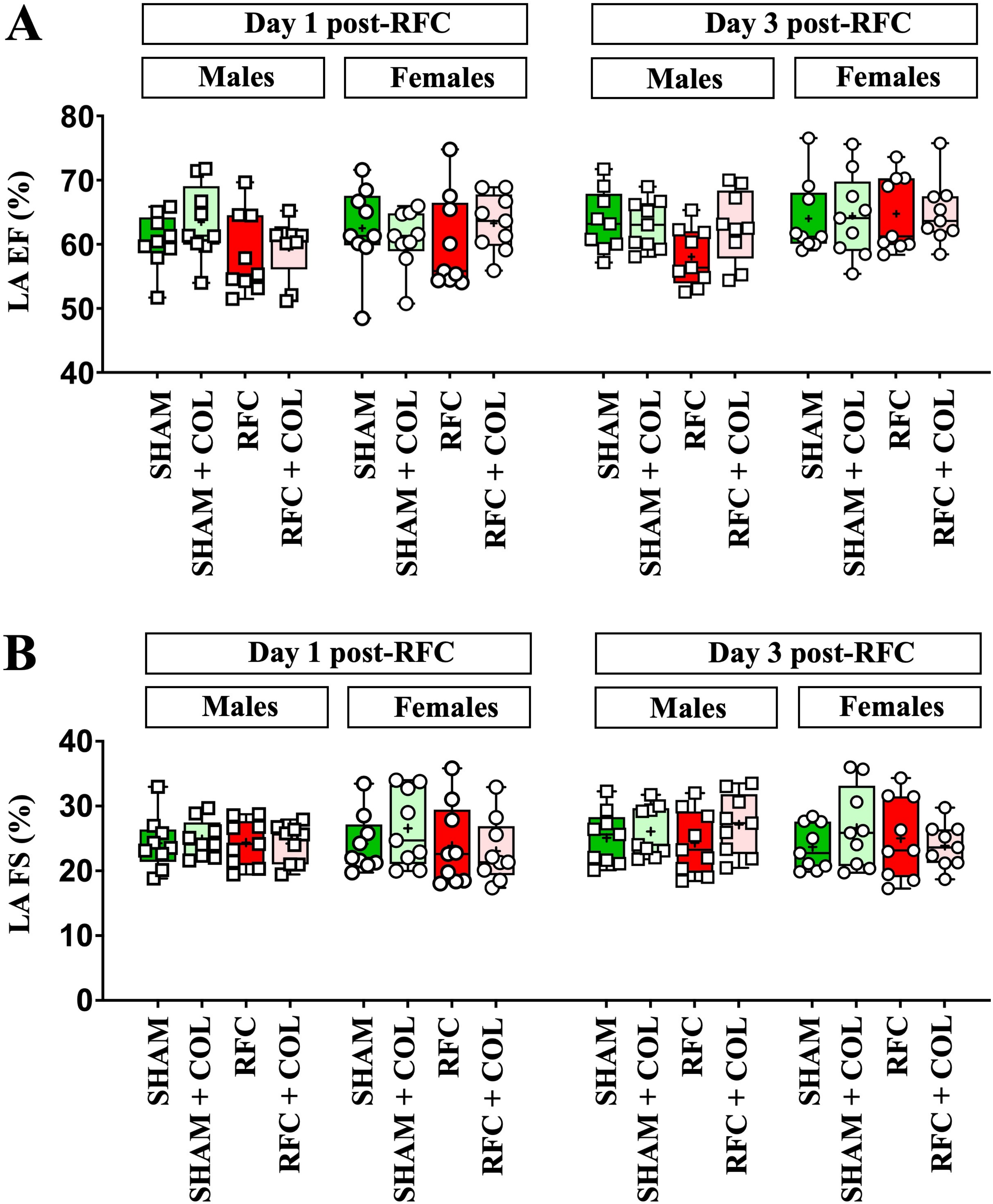
A
Day 1 post-RFC
Day 3 post-RFC
Males
Females
Males
Females
LA EF (%)
80
70
60
50
40
SHAM
SHAM + COL
RFC
RFC + COL
B
Day 1 post-RFC
Day 3 post-RFC
Males
Females
Males
Females
LA FS (%)
40
30
20
10
0
SHAM
SHAM + COL
RFC
RFC + COL

Online Figure S6. LA ejection fraction and fraction shortening. (**A**) Left atrial ejection fraction (LA EF) and (**B**) left atrial fraction shortening (LA FS) obtained by echocardiography and expressed in percent, in male and female sham, sham + COL, RFC, and RFC-COL rats at D1- and D3 post-RFC. *(Statistical analysis: A: Data were non-normally distributed as assessed by Shapiro-Wilk test. Mixed-effect model was applied to test statistical differences. B: Data were normally distributed as assessed by Shapiro-Wilk test, and two-way ANOVA was applied to test the differences. Homogeneity of variance was assessed using the Brown–Forsythe test, and Welch test was applied to confirm no significant differences between the groups. Each point results from an individual animal. n = 9 rats per group).*

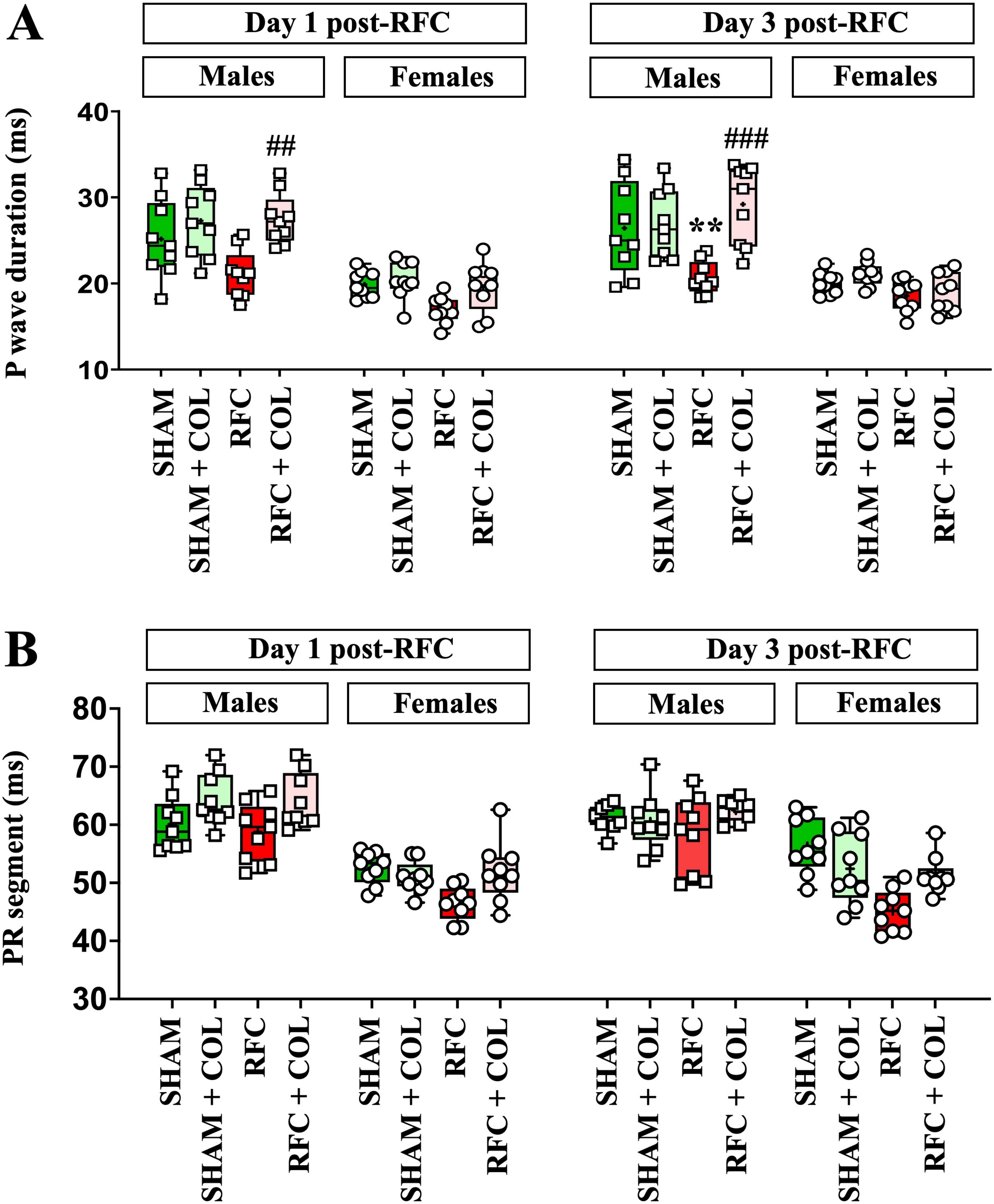

*** $p<0.01$ vs SHAM and ## $p<0.01$; ### $p<0.001$ vs RFC*

Online Figure S7. Electrocardiogram analyses (P and PR). ECG parameters (in ms) were measured by surface ECG analyses *in vivo*, including (**A**) P wave duration and (**B**) P-R interval in male and female sham, sham + COL, RFC, and RFC-COL rats at D1- and D3 post-RFC. *(Statistical analysis: Data were normally distributed as assessed by Shapiro-Wilk test. Groups were compared by two-way ANOVA followed by Tukey correction. In addition, Brown-Forsythe and Weich ANOVA tests were applied to evaluate homogeneity of variance and assess significance of differences between the groups. Each point results from an individual animal. n = 9 rats per group; ** $p<0.01$ vs SHAM and $^{\#\#}p<0.01$; $^{\#\#\#}p<0.001$ vs RFC).*

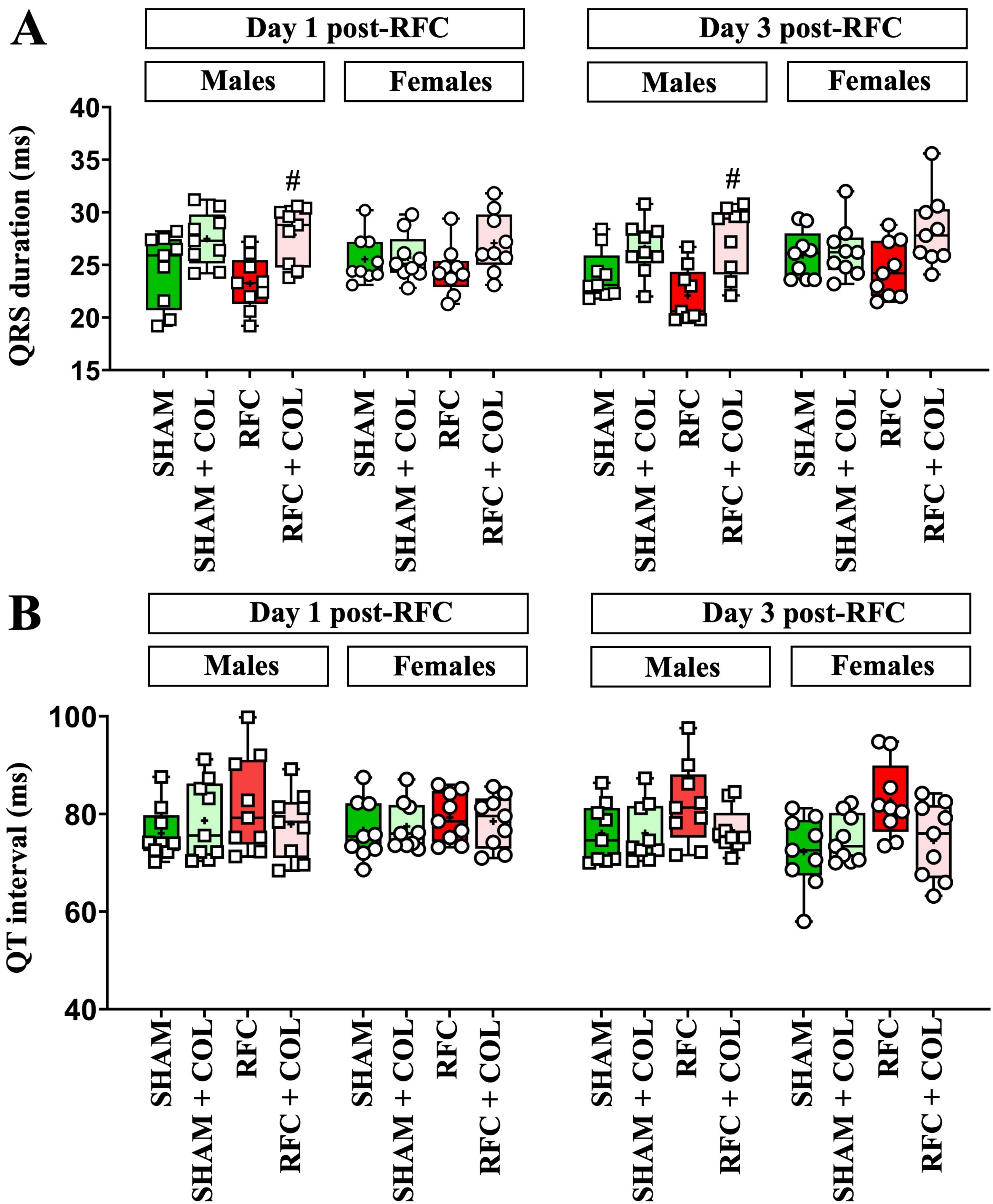
A
Day 1 post-RFC
Day 3 post-RFC
Males
Females
Males
Females
QRS duration (ms)
40
35
30
25
20
15
#
#
SHAM
SHAM + COL
RFC
RFC + COL
SHAM
SHAM + COL
RFC
RFC + COL
SHAM
SHAM + COL
RFC
RFC + COL
SHAM
SHAM + COL
RFC
RFC + COL
B
Day 1 post-RFC
Day 3 post-RFC
Males
Females
Males
Females
QT interval (ms)
100
80
60
40
SHAM
SHAM + COL
RFC
RFC + COL
SHAM
SHAM + COL
RFC
RFC + COL
SHAM
SHAM + COL
RFC
RFC + COL
SHAM
SHAM + COL
RFC
RFC + COL

# *p<0.05 vs RFC*

Online Figure S8. Electrocardiogram analyses (QRS and QT). ECG parameters (in ms) were measured by surface ECG analyses *in vivo*, including (**A**) QRS duration and (**B**) QT interval in male and female sham, sham + COL, RFC, and RFC-COL rats at D1- and D3 post-RFC. *(Statistical analysis: Data were normally distributed as assessed by Shapiro-Wilk test. Groups were compared by two-way ANOVA followed by Tukey correction. Brown-Forsythe and Welch ANOVA tests were applied to evaluate homogeneity of variance and assess significance of differences between the groups. Each point results from an individual animal. n = 9 rats per group;* $^{\#}p<0.05$ *vs RFC).*

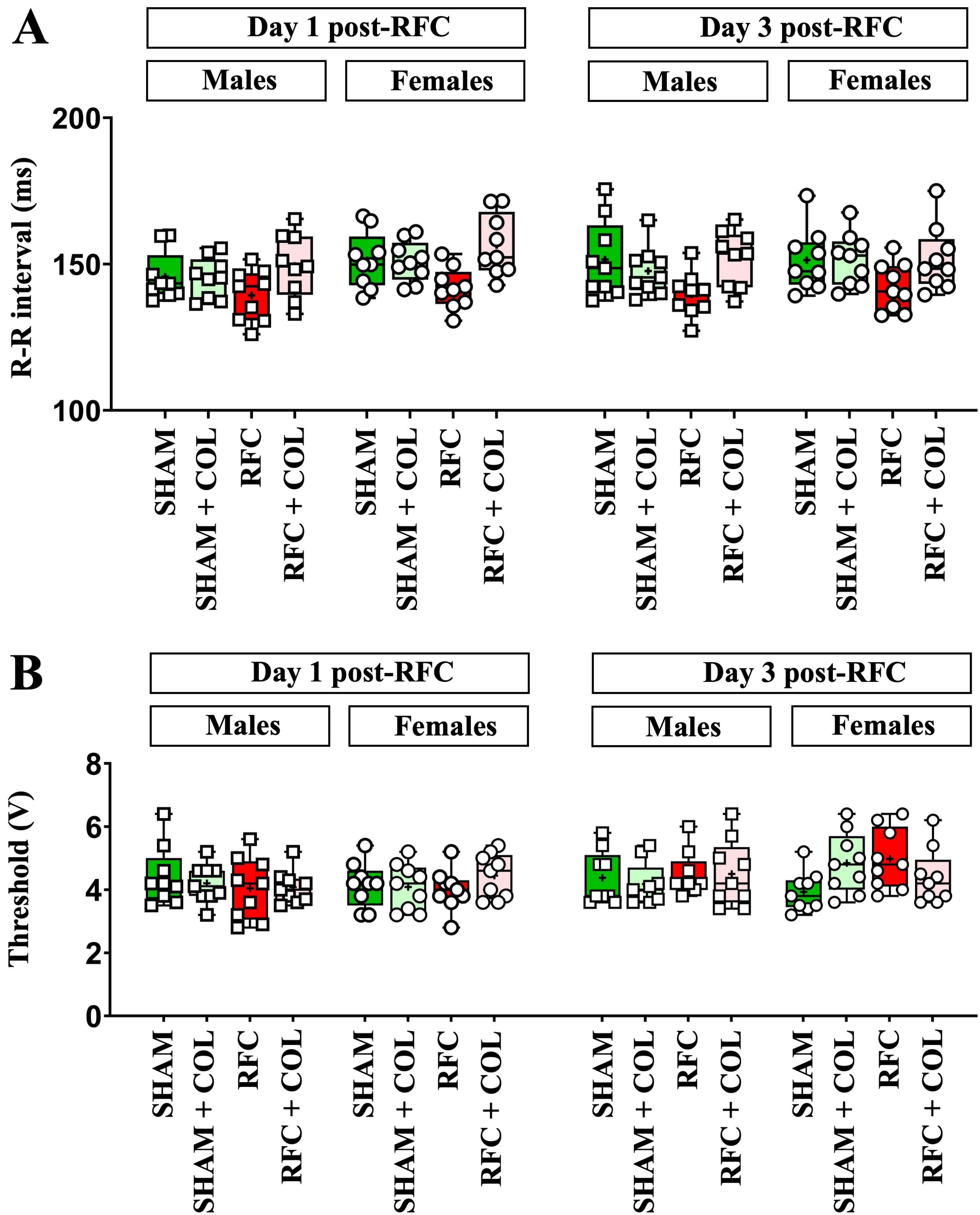
A
Day 1 post-RFC
Day 3 post-RFC
Males
Females
Males
Females
R-R interval (ms)
200
150
100
SHAM
SHAM + COL
RFC
RFC + COL
B
Day 1 post-RFC
Day 3 post-RFC
Males
Females
Males
Females
Threshold (V)
8
6
4
2
0
SHAM
SHAM + COL
RFC
RFC + COL

Online Figure S9. Electrocardiogram analyses. (**A**) R-R interval (in ms) was measured by surface ECG analyses *in vivo*, and (B) threshold was determined during electrophysiological study *in vivo*, in male and female sham, sham + COL, RFC, and RFC-COL rats at D1- and D3 post-RFC. *(Statistical analysis: Data were normally distributed as assessed by Shapiro-Wilk and tested by two-way ANOVA followed by Tukey correction. Homogeneity of variance was assessed using the Brown–Forsythe test, and Welch test was used to assess no significant differences between the groups. Each point results from an individual animal. n = 9 rats per group).*

RFC-induced Left Atrial Fibrosis in Scar area

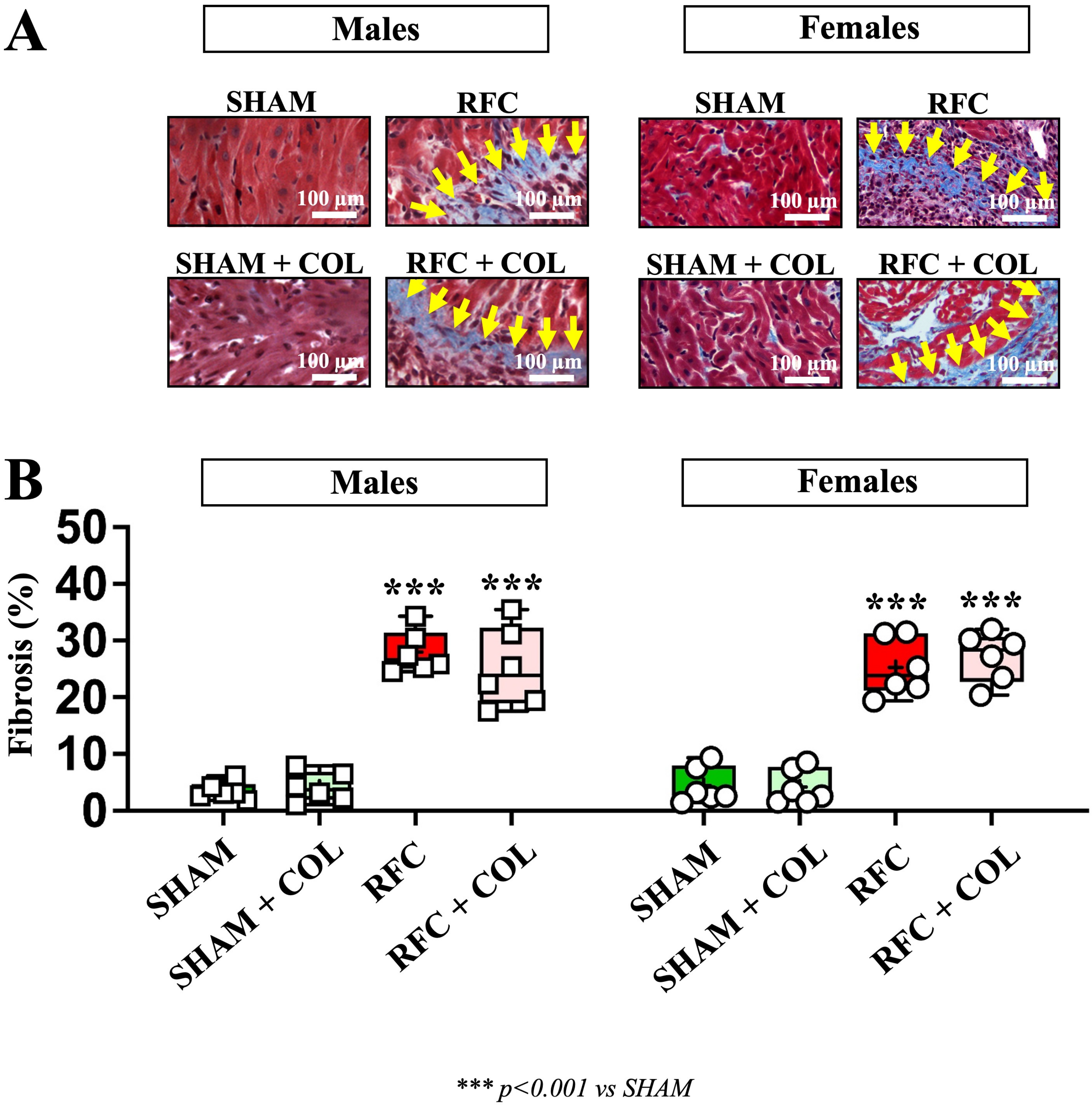

*** *p<0.001 vs SHAM*

Online Figure S10. Left Atrial Fibrosis in RFC scar area. (**A**) Representative transverse histological sections of the left atrium (LA) in RFC area, stained with Masson's Trichrome solution at D3, in male and female sham, sham + COL, RFC, and RFC + COL rats. Scale bars: 100 µm. (**B**) Quantification of the LA fibrous content expressed as a percent of the LA-surface where RFC was performed. *(Statistical analysis: B: Data were normally distributed as assessed by Shapiro-Wilk test. Two-way ANOVA was performed followed by Tukey correction. Brown-Forsythe and Welch ANOVA tests were applied to evaluate homoscedasticity and assess significance of differences between the groups. Each point represents results from an individual animal. n = 6 rats per group; ***p<0.001 vs SHAM).*
